# Bioactivity of Novel Colchicine, Colchiceine, and 10-Methylthiocolchicine Complexes with Lithium, Sodium, and Potassium Chlorides: Experimental and Theoretical Studies

**DOI:** 10.3390/ijms27072985

**Published:** 2026-03-25

**Authors:** Joanna Kurek, Patrycja Kwaśniewska-Sip, Wojciech Jankowski, Krzysztof Myszkowski, Grzegorz Cofta, Marcin Hoffmann, Marek Murias, Rafał Kurczab, Paweł Śliwa

**Affiliations:** 1 Department of Bioactive Products, Faculty of Chemistry, Adam Mickiewicz University, Uniwersytetu Poznańskiego 8, 61-614 Poznań, Poland; 2 Łukasiewicz Research Network-Poznań Institute of Technology, 6 Ewarysta Estkowskiego St., 61-755 Poznań, Poland; patrycja.kwasniewska-sip@pit.lukasiewicz.gov.pl; 3 Department of Quantum Chemistry, Faculty of Chemistry, Adam Mickiewicz University, Uniwersytetu Poznańskiego 8, 61-614 Poznań, Poland; w.jankowski@amu.edu.pl (W.J.); mmh@amu.edu.pl (M.H.); 4 Department of Toxicology, Poznań University of Medical Sciences, Rokietnicka 3, 60-806 Poznań, Poland; myszkowski.krzysztof.a@gmail.com (K.M.); marek.murias@ump.edu.pl (M.M.); 5 Institute of Chemical Wood Technology, University of Life Science, Wojska Polskiego 38/42, 60-037 Poznań, Poland; gcofta@gmail.com; 6 Maj Institute of Pharmacology, Polish Academy of Sciences, Smętna 12, 31-343 Kraków, Poland; rkurczab@gmail.com; 7 Faculty of Chemical Engineering and Technology, Cracow University of Technology, Warszawska 24, 31-155 Kraków, Poland; pawel.sliwa@pk.edu.pl

**Keywords:** colchicine, colchicine derivatives, cytotoxic activity, fungicidal activity, DFT calculations, molecular modeling

## Abstract

Complexes of colchicine, colchiceine, and 10-methylthiocolchicine with Li^+^, Na^+^, and K^+^ cations in the form of chlorides were synthesized and then subjected to spectral analysis, DFT theoretical studies, and molecular modeling. The values for water solubility and lipophilicity were also determined using various platforms; both factors are very important for determining the bioavailability of the tested compounds. These compounds were also tested for their fungicidal, herbicidal, insecticidal, and cytotoxic activities. Preliminary in silico studies showed that colchicine, colchiceine, 10-methylthio-colchicine, and their chloride complexes are inactive against selected fungi, weeds, and insects. Colchicine did not show antifungal properties in biological tests and was only active against *Aureobasidium pullulans*, as were its chloride complexes. The process of complexing colchiceine with metal cations in chloride salts significantly improved the antifungal potency against the selected species *A. pullulans* and *Chaetomium globosum*. The highest efficacy of colchiceine complexes was observed only against *A. pullulans* (MIC = 130 µg/mL) and *Ch. globosum* (MIC = 65 μg/mL). In contrast to the antifungal activity results, anticancer studies showed that 10-methylthiocolchicine complexes are more active against the SKOV-3 cell line (~IC50 = 2 nM) than colchicine or colchiceine. Molecular-modeling studies confirmed that lithium-coordinated compounds strongly stabilized the active ligand-tubulin complex, which may contribute to the observed cytotoxic activity.

## 1. Introduction

Colchicine **1** is a tropoloid alkaloid derived from *Colchicum autumnale* L. It has anti-inflammatory, anti-hypertrophic, and anti-inflammatory effects [1] and may be effective in relieving the symptoms of a gout attack when used in the early stages [2]. It has recently been used in the treatment of familial Mediterranean fever (FMF) [3]. In earlier studies on the binding activity of colchicine to proteins, it was found that this activity depends on the type of salts tested (Na_3_PO_4_, NaCl, sodium acetate, or sodium glutamate) in the formation of the colchicine-protein complex [4]. The maximum binding activity of colchicine to proteins was obtained at pH 6.7–6.8 [4]. Therefore, an increase in the NaCl concentration had no direct effect on the formation of the colchicine-protein complex, which was also observed in the presence of sodium acetate or sodium glutamate [4]. It is known that colchicine is sensitive to light and hydrolysis [5], and the main degradation product **1** is colchiceine **2** (Table 4). Colchicine **2** can be obtained by gentle hydrolysis with hydrochloric acid [6] or by isolating the naturally occurring alkaloid from *C. luteum Baker*, *C. kesselringii*, and *M. robusta* [7,8]. This compound is significantly less toxic than colchicine [9,10]. After the administration of colchicine **1**, colchicine **2** has been described as a metabolite in rats [11], but has not been observed in humans [12]. Mourelle et al. suggest that colchiceine **2** acts as an antioxidant and protective agent against lipid peroxidation in a rat model of liver damage [13]. Colchicine **1**, like other alkaloids, may act by blocking or activating specific receptors or ion channels in living organisms [14,15]. Its activity depends on its ability to form non-covalent complexes with macromolecules such as tubulin in microtubules [16]. 10-Methylthiocolchicine **3**, a semi-synthetic alkaloid, exhibits significant cytotoxic activity [17]. Biologically active and naturally occurring alkaloids, such as colchicine **1** and colchicine **2**, as well as 10-methylthiocolchicine **3** (Table 4), can form stable complexes, leading to the formation of more active compounds. In 1998, Mackay et al. obtained hydrated crystals of copper (II) colchicine [18].

The formation of complexes of colchicine and its derivatives with inorganic salts and their possible biological activity have not been thoroughly characterized. Several studies have been published on the complexation of colchicine **1**, colchiceine **2**, and 10-methylthiocolchicine **3** with various salts, showing that the complexes have better biological activity than the parent compounds [19,20,21,22,23,24,25,26,27].

We hypothesize that alkali metal coordination, particularly with Li^+^, enhances the tubulin-binding stability and cytotoxicity of colchicine derivatives by modifying their coordination modes and lipophilicity. All these facts prompted us to obtain and study the structure of the complexes of colchicine **1**, colchiceine **2**, and 10-methylthiocolchicine **3** with salts containing chloride anions and alkali cations: Li^+^, Na^+^, and K^+^. The aim of this study was to determine the biological activity, such as cytotoxicity against SKOV-3 ovarian cell lines and antifungal activity, of a series of complexes of colchicine **1**, colchiceine **2**, and 10-methylthiocolchicine **3** with the alkali metal cations (Li^+^, Na^+^, and K^+^) of chloride salts. Additionally, the molecular structures of colchicine **1**, colchiceine **2**, and 10-methylthiochicine **3** complexes with Li^+^, Na^+^, and K^+^ cations in methanol were theoretically evaluated using the DFT method. QM/MM and FMO calculations were used to investigate the interactions between 10-methylthiocolchicine and the lithium chloride complex **3-Li** and human tubulin. This approach has been successfully applied in our previous studies on colchicine and colchicine complexes with rubidium [26,27] and allowed us to discuss the role of ions in stabilizing complexes with the active site of β-tubulin.

## 2. Results and Discussion

The newly prepared complexes of colchicine **1**, colchiceine **2**, and 10-methylthiocolchicine **3** with the Li^+^, Na^+^, and K^+^ cations of chloride salts were characterized in detail using ESI MS, IR, ^13^C NMR, ^1^H NMR, UV-Vis, and elemental analysis; the data are provided in the Materials and Methods section and in the Supplementary Information.

A comparison of the changes in the ^1^H and ^13^C chemical shifts before and after the complexation of the salt by **1**, **2**, and **3** (Figures S1–S18) suggested that the metal cation was coordinated simultaneously by both oxygen atoms of the tropolone ring, the oxygen atoms of the acetamide, and the trimethoxybenzene moieties. The chemical structures of 1, 2, and 3 are given in Table 4.

**Figure 1:**
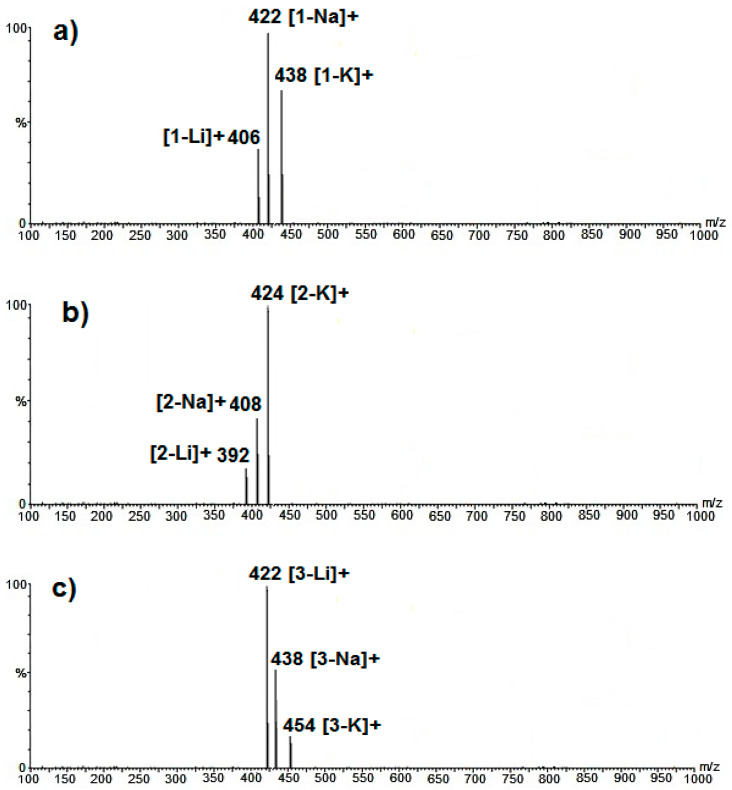
ESI MS competition experiments of mixtures with Li^+^, Na^+^, and K^+^ cations of chloride salts and the following—(**a**) colchicine **1**, (**b**) colchiceine **2**, and (**c**) 10-methylthiocolchicine **3**—in acetonitrile used in the 5:1:1:1:1:1 mutual ratio. ESI-MS competition spectra show preferential formation of 1-Na^+^, 2-K^+^, and 3-Li^+^ complexes, indicating cation selectivity.

The ESI MS studies indicate that the stoichiometry of the complexes is L-M^+^, L-2M^+^-X-, 2L-M^+^, 3L-M^+^, and 2L-2M^+^-X^−^, where **L** (ligand) is **1** or **2** or **3**, M^+^ is a metal cation, and X^−^ is an anion, Table 1 and the ESI MS spectra for the separate ligands and respective cations are given in Supplementary Information (Figures S22–S24). The relative intensities obtained in competitive experiments (Figure 1) showed that the formation of 1:1 complexes between **1** and Na^+^, **2** and K^+^, and **3** and Li^+^ was the most preferred. 

Competitive ESI MS experiments of mixtures with the Li^+^, Na^+^, and K^+^ cations of chloride salts with the corresponding alkaloids—colchicine, colchicine, and 10-methylthio-colchicine—are shown in Table 1.

Based on the ESI MS studies, it was found that 10-methylthiocolchicine **3** can form stable complexes with lithium in two different stoichiometries: 1:1:1 and 2:1.

**Figure 2:**
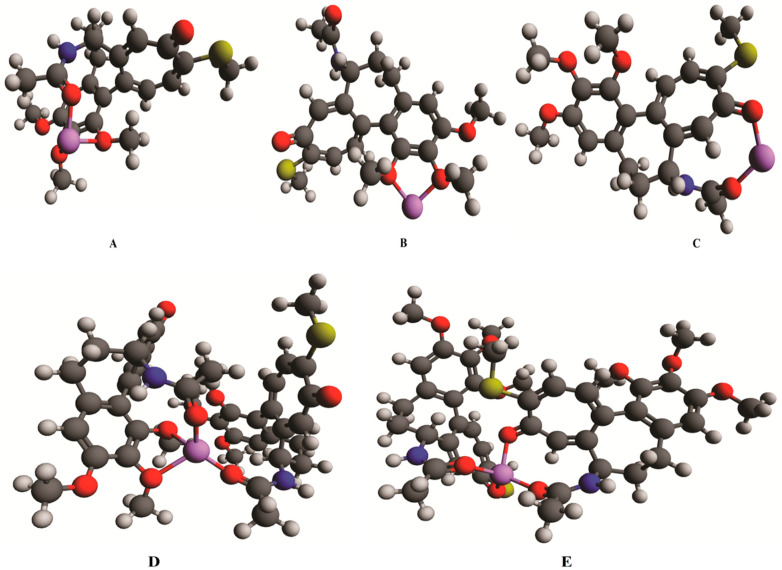
Optimized structures with 1:1 stoichiometry (**A**–**C**) and with 2:1 stoichiometry (**D**,**E**).

Seven interaction schemes of 10-methylthiocolchicine complexes with lithium based on the possible interactions described [21,24] were subjected to further computational studies. Only five will be described; even though it seemed unlikely that we would be able to obtain complexes in which one or both (stoichiometry 1:1:1 or 2:1) molecules of 10-methylthiocolchicine were coordinated via S1 sulfur and O5 oxygen atoms, we examined those complexes. Preliminary calculations showed that such interactions were not likely to occur and will not be considered. In the initial interaction schemes of the Type A complex with 1:1 stoichiometry, the 10-methylthiocolchicine molecule was coordinated via O1, O2 and O4 oxygen atoms; in Type B, via O1 and O2 oxygen atoms; and, in Type C, via O4 and O5 oxygen atoms. In the initial interaction schemes of the Type D complexes with 2:1 stoichiometry, both 10-methylthiocolchicine molecules were coordinated via O1, O2, and O4; and, in Type E, via O4 and O5 (Figure 2).

Table 2 shows the interaction energy values for each type of the studied complexes in vacuum and methanol (the solvent used for synthesis). An extended version of Table 2 with counterpoise energy, BSSE, and the sum of monomer energy is included in Supplementary Information, Table S5.

For 10-methylthiocolchicine complexes with 1:1 stoichiometry, the Type C complex in which 10-methylthiocolchicine coordinated with lithium via O4 and O5 oxygen atoms had the most energetically favored interaction energy, in vacuum and methanol. The energy of this complex differed from that of other complexes by 7.7 kcal/mol for Type A and 35.0 kcal/mol for Type B complexes in vacuum and 1.4 kcal/mol for Type A and 7.3 kcal/mol for Type B complexes in methanol. For 2:1 stoichiometry complexes, the most energetically favored structures were obtained for Type E complexes (in vacuum and methanol) in which both molecules of 10-methylthiocolchicine **3** coordinated with Li^+^ via O4 and O5 oxygen atoms. The interaction energy of this complex was more favored than for the Type D complex only by 7.9 kcal/mol in vacuum and 0.3 kcal/mol in methanol. The optimized structures of the 1:1 (A–C) and 2:1 (D,E) stoichiometry complexes are shown in Figure 2. The atomic coordinates of the complexes are included in the Supplementary Materials, Tables S5–S7. For the Type D complex with 2:1 stoichiometry, the initially selected mode of coordination changed after optimization. In this structure, one 10-methylthiocolchicine coordinates via the O1, O2, and O4 oxygen atoms and the other only via the O4 oxygen atom. Selected interatomic distances and calculated Mulliken point charges are listed in Table S6.

In the most energetically favored complex of 1:1 stoichiometry, the calculated Mulliken partial charge on the lithium cation was 0.630 and −0.520 for the O4 atom and −0.471 for the O5 atom on the coordinating atoms of 10-methylthiocolchicine. For the most energetically favored complex, Type E with 2:1 stoichiometry, the Mulliken charge on the lithium cation was −0.092 and −0.488 for O4a, −0.265 for O5a, −0.410 for O4b, and −0.426 for O5b on the coordinating atoms of 10-methyltiocolchicine molecules. In all of the investigated complexes with 10-methylthiocolchicine coordinating via O4 oxygen atoms, we observed the shortest distances (shorter than the distances measured for the other coordinating atoms in complexes) between the coordinating atom and Li^+^. It was observed that the calculated Mulliken charges for those coordinating atoms were lower than those calculated for others.

To validate the reliability of the M06/6-31+g(d,p) level employed for geometry optimization and interaction energy calculations, we performed comparative single-point calculations on the optimized geometries of 1:1 stoichiometry complexes at a higher, widely accepted wB97XD/Def2TZVPP [28,29] method and basis set. The results of those calculations are gathered in Supplementary Information Table S6. A comparison of the interaction energies shows that, between those two methods, the interaction energy differed from 1.2 to 2.2 kcal/mol. Importantly, the energetic ordering of the complexes is fully preserved: Type C remains the most favored, followed by Type A, with Type B clearly the least stable. In conclusion, the close agreement with the higher-level wB97XD/Def2TZVPP reference confirms that the M06/6-31+g(d,p) level is of sufficient quality for the purposes of this work.

Previous studies reported that some complexes of colchicine **1**, colchiceine **2**, and 10-methylthiocolchicine **3** exhibited fungicidal activity comparable to selected reference fungicides, such as IPBC or chalcone [21,23,25]. In the present study, we evaluated whether complexes of colchicine **1**, colchiceine **2**, and 10-methylthiocolchicine **3** with chlorides of alkali metal ions (Li^+^, Na^+^, and K^+^) had some fungicidal activity against previously selected microfungal species. The bioassay results of colchicine **1**, colchiceine **2**, 10-methylthiolchicine **3**, their respective salts, and their complexes against microfungi are listed in Supplementary Information, Table S2, with the results for the minimal fungicidal concentration (MFC). The results of the bioassays for the minimal inhibitory concentration (MIC) are listed in Table 3, demonstrating a limited and highly selective activity profile of the investigated complexes. Colchicine complexes **1-LiCl**, **1-NaCl**, and **1-KCl** were inactive against all tested microfungal species.

Complexes of 10-methylthiocolchicine **3-LiCl**, **3-NaCl**, and **3-KCl** showed only weak and species-specific activity restricted to *A. pullulans* and *P. cyclopium*, with no detectable effects against the remaining strains. Among all tested systems, only the colchiceine **2-LiCl**, **2-NaCl**, and **2-KCl** complexes showed measurable antifungal activity, which was confined to a small subset of fungi, including *P. funiculosum*, *A. pullulans*, and *Ch. globosum*. The lowest MIC values were observed for *A. pullulans* and *Ch. globosum***,** at 65 [μg/mL] and 130 [μg/mL], respectively. The differences in the activity of the tested compounds between *Ch. globosum* and *A. pullulans* and the other tested fungi may be due to their physiology. *Ch. globosum* is classified as an ascomycete fungus [26,27], while *A. pullulans* is classified as a yeast-like fungus [30,31], while the other tested fungi are molds. However, this activity remains restricted to a narrow spectrum of organisms. Although, in these isolated cases, the activity exceeded that of chalcone, such comparisons should be interpreted with caution and should not be generalized to imply overall fungicidal efficacy. The observed trend of increasing activity with decreasing cation size (K^+^ > Na^+^ > Li^+^) for *A. pullulans* is qualitative in nature and limited to a single species, which precludes broader mechanistic conclusions. Overall, the data suggest that the investigated complexes exhibit, at most, a niche-specific antifungal potential, and their relevance as broad-spectrum fungicidal agents remains limited.

The compounds colchicine **1**, colchiceine **2**, and 10-methylthio-colchicine **3** and their complexes were tested as herbicides, insecticides, and fungicides by the Open Innovation Platform Agro (BASF). The aim was to determine whether the compounds tested are active against the following fungal strains: *Phytophthora infestans*, *Zymoseptoria tritici*, *Botrytis cinerea*, *Fusarium culmorum* and against the insects: boll weevil (*Anthonomus grandis*), Mediterranean fruitfly (*Ceratitis capitata*), vetch aphid (*Megoura viciae*), *Caenorhabditis elegans*, tobacco budworm (*Heliothis virescens*), green peach aphid (*Myzus persicae*), Greenhouse Whitefly (*Trialeurodes vaporariorum*), and yellow fever mosquito (*Aedes aegypti*), as well as weeds such as shepherd’s purse (*Capsella*), creeping bentgrass (*Agrostis stolonifera*), and annual meadow grass (*Poa annua*). The compounds were first pre-screened according to BASF procedures. Colchicine **1**, colchiceine **2**, 10-methylthiocolchicine **3**, and their complexes were tested in silico in the company’s system as potential biologically active molecules, but they did not show such activity and were rejected from further biological testing.

In order to evaluate the cytotoxic activity of the synthesized complexes, tests in the SKOV-3 ovarian cancer cell line were performed and the IC_50_ values are given in Table 4.

All salts were also tested for their cytotoxic effect on human SKOV-3 ovarian cancer cells. The results showed that the tested salts themselves had no effect on SKOV-3 cells at the concentrations used in the experiment (0–10 µM). In cytotoxicity studies, the tested compounds were dissolved in DMSO (final concentration: 0.1%). Doxorubicin was used as a positive control and DMSO alone as a negative control. The cytotoxic activity of the obtained complexes was tested on SKOV-3 ovarian cancer cell cultures using the MTT assay. The cytotoxic effects of the tested compounds and their corresponding salts (LiCl, NaCl, and KCl) on SKOV-3 cells assessed using the MTT assay are shown on Figure S28. The compounds tested showed potent cytotoxic activity against the cell lines. Compound **3** was more potent than colchicine **1**, but it was found, after the complexation process with inorganic salts (Li^+^, Na^+^, and K^+^ chlorides), that the activity of those complexes against cancer cells increased.

Because the investigated compounds belong to the colchicine-site tubulin inhibitor scaffold, the most informative comparison in the present work is the internal, mechanism-consistent series tested under identical conditions (1 vs. 3 vs. 3–MCl; Table 4). Doxorubicin was used only as a routine positive control to confirm the performance of the MTT readout and should not be interpreted as a mechanistic reference (DNA intercalation vs. tubulin binding). Thus, the main conclusion regarding the enhanced potency upon metal coordination is based on direct within-study comparisons within the same pharmacophore family.

To understand why some compounds showed higher activity than others, in silico tests were conducted to predict key parameters such as lipophilicity, hydrophobicity (water solubility), and toxicity. These parameters are crucial for understanding the action of compounds at the cellular level and their interaction with pathogens such as the microfungi species mentioned above.

The SwissADME online tool was used to predict the water solubility of the tested compounds (details are provided in SI). Water solubility is a key physical property in medicinal chemistry, but not only there, as it affects the distribution and absorption of biologically active compounds in living cells, tissues, organisms, and the environment. It should be noted that the optimal distribution and absorption of these compounds require good water solubility. Compounds can be classified based on their solubility value (LogS). Compounds with high solubility have solubility values of 0 or higher, while those in the range of 0 to −2 are considered soluble.

Compounds with solubility values between −2 and −4 are poorly soluble, and those with solubility values below −4 are insoluble. Table 5 shows the LogS values obtained using the ESOL, Ali, and SILICOS-IT methods. According to the data in Table 5, all 12 compounds are soluble or moderately soluble, and some of them are slightly soluble. For comparison, the water solubility of compounds that are standards used in biological tests has also been added. Compound **3-Li**, the most active in the cytotoxicity test, is less soluble than the starting compounds **1** and **3** (colchicine and 10-methylthio-colchicine, respectively).

The role of lipophilicity in drug discovery and design is a critical one. Lipophilicity is a key physicochemical property that plays a crucial role in determining ADMET (absorption, distribution, metabolism, excretion, and toxicity) properties and the overall suitability of drug candidates. A growing body of evidence suggests that maintaining physicochemical properties, such as lipophilicity, within a specific optimal range can improve the quality of a compound and increase the likelihood of therapeutic success. Lipophilicity is a significant factor that modulates molecular interactions, resulting in changes in ligand-receptor binding or the enzyme inhibition potency, but also as a property that facilitates passive drug transport [32]. LogP is essential, but predicting it accurately is difficult, especially for large, flexible, drug-like molecules. The calculated LogP values (Table 6) of the tested compounds, **1-LiCl**, **1-NaCl**, **1-KCl**, **2-LiCl**, **2-NaCl**, **2-KCl**, **3-LiCl**, **3-NaCl**, and **3-KCl**, showed that the lipophilicity of the respective compounds increases after the complexation process in comparison to **1**, **2**, and **3**, respectively. The lipophilicity of all 12 compounds and standard compounds (doxorubicin, chalcone, and IPBC) was predicted using three different web tools (MolInspiration, SwissADME, and Protox II) to enable a data comparison and the results of the LogP values of the tested compounds are summarized in Table 6. There is no perfect method to obtain LogP values. It is connected with the platform used for calculations and many other factors. The SwissADME web tool offers five methods (iLOGP, XLOGP3, WLOGP, MLOGP, and SILICOS-IT) to predict the lipophilicity of compounds, as well as a Consensus LogP parameter, which is the average value of all five predictions. Consensus models (averaging predictions from several reliable methods) gave the best accuracy overall, indicating an increase in lipophilicity for the derivatives compared to the parent compound **1**. Specifically, compounds **3**, **3-LiCl**, **3-NaCl**, and **3-KCl** have a LogP value of 2.17 (obtained from MolInspiration), while compound **1** has a logP value of 1.10, indicating a significantly higher lipophilicity than **1**. The LogP values calculated by the Protox II platform (LogS) were slightly higher but followed the same trend. The LogP values obtained varied depending on the computational platform used and also the computational methods [33,34]. Compounds with LogP values between 1 and 3 often have better bioavailability after oral administration (according to Lipinski’s rule of five), which means that the respective compound has a moderate solubility and permeability, and is often ideal for use in drugs. Considering the values obtained in the context of Lipinski’s rule, the most favorable results were obtained for the following platforms: MolInspiration and SwissADME (MLOGP and Consensus Log P). In contrast, the values obtained for SwissADME and LOGP and SILI-COS-IT were too low, and those for SwissADME (XLOGP3 and WLOGP) and Protox II were too high. In general, if a compound has a LogP value above 3, it binds strongly to proteins, easily penetrates cell membranes, has a long half-life, can accumulate in adipose tissue, and requires metabolism before excretion. If a compound has a LogP value significantly above 3, it poses a risk of bioaccumulation and toxicity if it is too lipophilic (above 5). Although the indicated LogP values are lower or higher than the range of 1–3, they are still relevant. The optimal value according to Lipinski’s rule for the most favorable bioavailability (optimal zone for most ADMET = absorption, distribution, metabolism, excretion, and toxicity properties) is in the range pf 1–3, but those with values up to 5, as well as those between 1–0, still comply with this rule, but are more hydrophobic and more hydrophilic, respectively. This means that the tested compounds are largely characterized by the potentially optimal bioavailability, while some of them are slightly worse, but their LogP values fall within the range indicated by Lipinski’s rule.

As a part of the MolInspiration tool, we used Galaxy Visualizer 3D to display the molecular lipophilicity potential (MLP) on the molecular surface (see Figure S25). According to the PROTOX II prediction, **3-LiCl**, **3-NaCl**, and **3-KCl** are active as cytotoxic agents (Table S4).

The increase in the lipophilicity of all the complexes in comparison to the respective alkaloids alone presumably causes increased biological activity in cytotoxic tests against SKOV-3 cancer cell lines. The impact of the water solubility of the complexes on their cytotoxic activity (IC_50_ values) is shown in Figure S29. While increased lipophilicity correlates with higher cytotoxicity, causality requires further mechanistic studies.

The cytotoxicity results (IC_50_ values; Table 4) indicate that complexes of 10-methylthiocolchicine exhibit the highest activity among the tested compounds. To elucidate the molecular basis of this enhanced activity, advanced theoretical studies were performed using the lithium-containing complex (**3–Li**) as a representative model of alkali metal coordination within the colchicine-binding site of human β-tubulin. In parallel, DFT calculations in methanol were carried out to identify the preferred coordination modes of the lithium cation with a single 10-methylthiocolchicine molecule, which were subsequently considered in the QM/MM and FMO/EDA analyses. The computational analysis was designed as a representative mechanistic case study for the most cytotoxic class, i.e., 10-methylthiocolchicine complexes. The detailed ONIOM/FMO modeling of **3-Li** is therefore intended to rationalize the binding features of the 3-complex class as a whole, noting that the Na^+^ and K^+^ analogues show a comparable low-nM cytotoxicity (Table 4).

**Figure 3:**
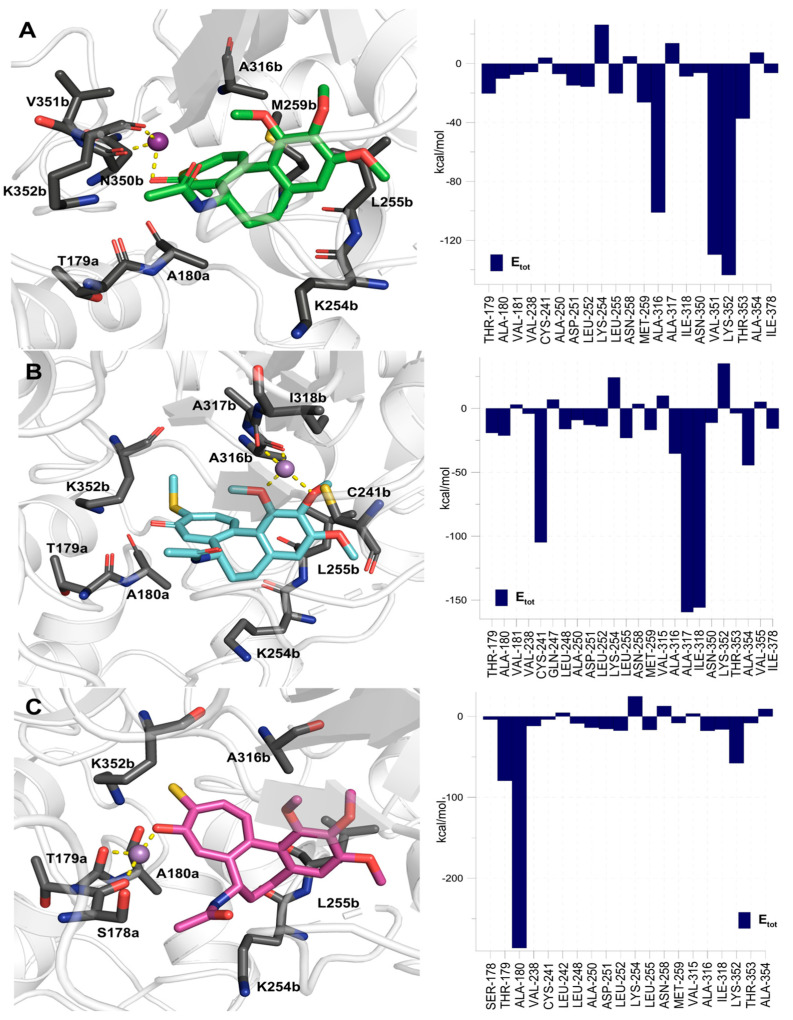
ONIOM-optimized complexes and FMO energy profiles of three **3-Li** conformers within the colchicine-binding site of the human α/β-tubulin dimer (PDB ID: 5EYP). (**A**) conformer A; Li^+^ with tubulin binding site, (**B**) conformer B: Li^+^ stably clenched between the two methoxy oxygens, and (**C**) conformer C: Li^+^ bond with the acetamide substituent. Residues are labeled according to their sequence number, with the letters “a” and “b” indicating residues from the α- and β-tubulin subunits, respectively. Interactions of the lithium cation are marked as yellow dotted lines. All hydrogen atoms are omitted.

Full QM/MM optimization also resulted in three possible conformations of the complexes in the binding pocket of human β-tubulin (Figure 3). In addition, full QM/MM optimization showed three possible arrangements of the lithium ion in the binding pocket of human β-tubulin (Figure 3). Despite that, the position of molecule **3** was similar in each case and all key interactions for tubulin polymerization inhibitors, e.g., with residues K352b, A316b, K254b, A180a, and T179a, were identified [25,35,36,37]. The lithium cation coordination site to **3** in complexes B and C was preserved as in the optimized DFT results. In complex A, the Li^+^ coordination pattern was reorganized as a result of incorporation into the tubulin binding site, and that position was additionally stabilized by interactions with the carbonyl oxygen from residues N350b and K352b (Figure 3A). This binding mode of the lithium cation was analogous to that for rubidium iodide, observed in the previous report [38]. In the conformation of B, lithium was very stably clenched between the two methoxy oxides of molecule **3**, and such coordination could be additionally forced by interactions with A317b and I318b (Figure 3B).

In the case of complex C, the Li^+^ bond with the acetamide substituent, found in the DFT calculations, was lost in favor of interactions with residues S178a, T179a, and A180a of tubulin.

In the second stage, the energy single-point calculations of the ligand-protein stabilization were performed using the FMO-EDA approach (Figure 3). It should be noted that MP2 calculations performed with moderate basis sets may exhibit limited quantitative accuracy; therefore, the FMO-MP2 interaction energies reported herein are interpreted primarily in a comparative and qualitative manner. The comparison of the total interaction energy showed that the B arrangement of the lithium cation was energetically favored within the applied computational model (the sum of E_tot_ was −504.4, 580.0, and 511.6 kcal/mol for A, B, and C, respectively). The FMO-EDA results (Figure S29 in Supplementary) reveal that the binding of the 3–Li complex is not governed by dispersion-driven hydrophobic contacts alone, but rather by a pronounced electrostatic and charge-transfer component associated with Li^+^ coordination. Conformer B benefits from stronger polarization and charge-transfer interactions with key residues of the colchicine site, which outweigh the increased exchange repulsion observed for this tightly packed arrangement. This energetic balance rationalizes the preference for binding mode B at the QM/MM level. However, it should be considered that the interaction energies discussed herein reflect the relative binding strengths at the applied level of theory and should not be interpreted as absolute measures of thermodynamic stability. While a rigorous thermodynamic treatment would require the inclusion of ligand deformation (strain) energy, the primary objective of this study is to characterize the physical nature of the interactions stabilizing the ligand-tubulin complex and to evaluate the specific role of the coordinated metal cation in this stabilization.

The dihedral angle ω(C1-C1a-C12a-C12) between the A and C rings, crucial for binding between these alkaloids and tubulins for colchicine, is ω ~ 53° [12,38]. For the colchicine–Na complex, the ω value in molecular modeling was 47° [25]. For the 10-methylthiocolchicine-Rb complex, the ω value in molecular modeling was 43° [35]. For the 10-methylthiocolchicine-Li complex, the dihedral angle values in molecular modeling were **3-Li^+^** A 66°, **3-Li^+^** B 72°, and **3-Li^+^** C 57°, respectively, and was found to differ substantially to colchicine or its heavier alkali metal analogues. In particular, the most stable binding mode (conformer B) adopts a more open geometry (ω ≈ 72°), which can be attributed to the strong coordination of the Li^+^ cation. This coordination rigidifies the ligand framework and enforces a distinct relative orientation of the aromatic rings. Importantly, despite this geometric deviation, the key interactions characteristic of colchicine-site inhibitors are preserved. The altered dihedral angle, therefore, reflects an adaptive binding mode rather than a loss of complementarity and may contribute to enhanced electrostatic stabilization within the binding pocket, consistent with the observed binding preference and cytotoxic activity of the **3-Li** complex.

## 3. Materials and Methods

### 3.1. Materials

Colchicine **1**, a natural (-)-(a*R*,7*S*) colchicine isomer, was obtained commercially from Sigma Aldrich. Colchiceine **2** was obtained from colchicine [7]. 10-Methylthiocolchicine **3** was obtained from colchicine using a method described in [17]. The salts (LiCl, NaCl, and KCl) were purchased from Sigma-Aldrich Poland and used without purification. The solvents used for the synthesis were purified using standard methods or were pure for analysis (ChemPur, Poland).

#### 3.1.1. Preparation of Complexes

Colchicine complexes: [Li(C_22_H_25_NO_6_)Cl] **1-LiCl**, [Na(C_22_H_25_NO_6_)Cl] **1-NaCl**, [K(C_22_H_25_NO_6_)Cl] **1-KCl**. The complexes were prepared from colchicine **1** (100 mg, 0.25 mmol) and lithium chloride (10.6 mg, 0.25 mM), sodium chloride (27 mg, 0.25 mM), and potassium chloride (27 mg, 0.25 mM), respectively.

Colchiceine complexes: [Li(C_21_H_23_NO_6_)Cl] **2-LiCl**, [Na(C_21_H_23_NO_6_)Cl] **2-NaCl**, [K(C_21_H_23_NO_6_)Cl] **2-KCl**. The complexes were prepared from colchiceine **2** (100 mg, 0.25 mM) and lithium chloride (11 mg, 0.25 mM), sodium chloride (27 mg, 0.25 mM), and potassium chloride (27 mg, 0.25 mM), respectively.

10-Methylthiocolchicine complexes: [Li(C_22_H_25_NO_5_S)Cl] **3-LiCl**, [Na(C_22_H_25_NO_5_S)Cl] **3-NaCl**, [K(C_22_H_25_NO_5_S)Cl] **3-KCl**. The complexes were prepared from 10-methylthiocolchicine **3** (100 mg, 0.25 mM) and lithium chloride (10 mg, 0.25 mM), sodium chloride (14 mg, 0.25 mM), and potassium chloride (18 mg, 0.25 mM), respectively.

The colchicine **1**, colchiceine **2**, and 10-methylthiocolchicine **3** complexes with salts (LiCl, NaCl, and KCl from Aldrich) were obtained by dissolving the respective salts and colchicine **1**, colchiceine **2**, and 10-methylthiocolchicine **3**, respectively, in a 1:1 ratio in methanol. Crystalline products were obtained by slow evaporation of the solvent. ^1^H NMR and ^13^C NMR spectra of each obtained complexes are given in Supplementary Information.

**Scheme 1:**
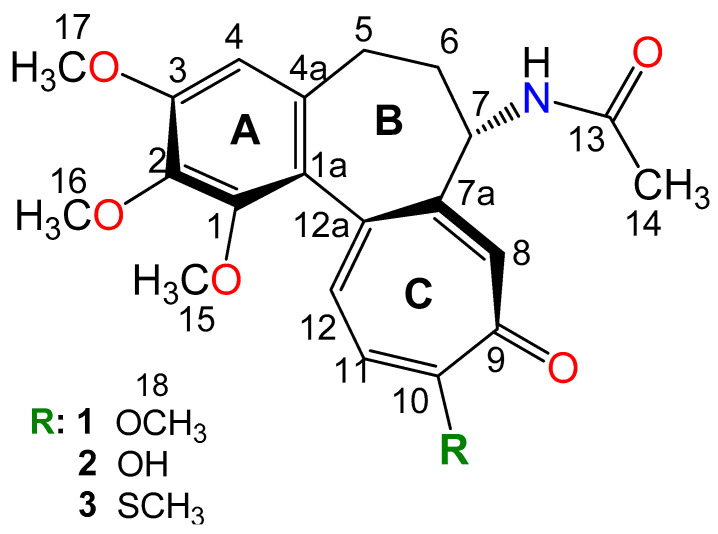
Carbon atom numbering of colchicine **1,** colchiceine **2**, and 10-methylthiocolchicine **3.**

The carbon atom numbering of colchicine **1**, colchiceine **2**, and 10-methylthiocolchicine **3** is shown in Scheme 1.

Complex **1-LiCl**: Yield 96%, 107 mg; m.p. = 125–127 °C, UV (MeOH, λ_max_, [cm^−1^]): 351, 245, ^1^H NMR (CD_3_CN, 300.075 MHz, 25 °C, ppm): 6.55 (1H, CH, s, C4), 2.42 (2H, CH_2_, C5), 1.96 (2H, CH_2_, C6), 4.56 (1H, CH, C7), 7.59 (1H, CH, s, C8), 6.91 (1H, CH, d, *J* = 10.6 Hz, C11), 7.37 (1H, CH, d, *J* = 10.6 Hz, C12), 3.67 (3H, CH_3_, s, C-15), 3.92 (3H, CH_3_, s, C-16), 3.95 (3H, CH_3_, s, C17), 4.02 (3H, CH_3_, s, C18), 1.96 (3H, CH_3_, s, C14), 8.14 (1H, NH, d, *J* = 7.36 Hz); ^13^C NMR (75 MHz, CD_3_CN, TMS, 25 °C, ppm): 151.2 (C1), 125.6 (C1a), 141.6 (C2), 153.5 (C3), 107.3 (C4), 136.9 (C4a), 29.8 (C5), 36.4 (C6), 52.7 (C7), 152.5 (C7a), 130.5 (C8), 179.5 (C9), 164.0 (C10), 112.9 (C11), 135.6 (C12), 134.2 (C12a), 170.0 (C13), 22.8 (C14), 77.2 (C15), 76.6 (C16), 56.1 (C17), 56.4 (C18). FT IR (KBr, cm^−1^): 3391, 3252, 3049, 2938, 2839, 1658 ν_(CO)_, 1612 ν_(CO)_, 1543, 1589, 1488, 1255, 1139, 1093. Anal. El. analysis (calcd., found for C_22_H_25_NO_6_·LiCl·H_2_O): C (57.18, 57.14), H (5.82, 5.79), N (3.04, 3.01).

Complex **1-NaCl**: Yield 97%, 110 mg; m.p. = 106–108 °C, UV (MeOH, λ_max_, [cm^−1^]): 351, 244, ^1^H NMR (CD_3_CN, 300.075 MHz, 25 °C, ppm): 6.7 (1H, CH, s, C4), 2.3, 2.56 (2H, CH_2_, C5), 2.11, 2.24 (2H, CH_2_, C6), 4.36 (1H, CH, C7), 7.19 (1H, CH, s, C8), 6.92 (1H, CH, d, *J* = 10.72 Hz, C11), 7.15 (1H, CH, d, *J* = 10.72 Hz, C12), 3.61 (3H, CH_3_, s, C15), 3.86 (3H, CH_3_, s, C16), 3.83 (3H, CH_3_, s, C17), 3.90 (3H, CH_3_, s, C18), 1.94 (3H, CH_3_, s, C14), 7.21 (1H, NH, d, *J* = 7.36 Hz); ^13^C NMR (75 MHz, CD_3_CN, TMS, 25 °C, ppm). 151.71 (C1), 126.57 (C1a), 142.11 (C2), 154.33 (C3), 108.53 (C4), 136.54 (C4a), 30.25 (C5), 36.83 (C6), 52.93 (C7), 151.82 (C7a), 131.32 (C8), 179.62 (C9), 164.88 (C10), 112.92 (C11), 135.57 (C12), 135.51 (C12a), 170.12 (C13), 22.72 (C14), 61.61 (C15), 61.39 (C16), 56.58 (C17), 56.76 (C18). FT IR (KBr, cm^−1^): 3441, 3359, 3125, 2998, 2970, 2938, 2832, 1650 ν_(CO)_, 1610 ν_(CO)_, 1552, 1490, 1253, 1135, 1093. Anal. El. analysis (calcd., found for C_22_H_25_NO_6_NaCl·0.5H_2_O): C (56.77, 56.73), H (6.42, 6.40), N (2.99, 2.98).

Complex **1-KCl**: Yield 97%, 114 mg; m.p. = 120–122 °C, UV (MeOH, λ_max_, [cm^−1^]): 352, 244, ^1^H NMR (CD_3_CN, 300.075 MHz, 25 °C, ppm): 6.69 (1H, CH, s, C4), 2.33, 2.56 (2H, CH_2_, C5), 2.11, 2.24 (2H, CH_2_, C6), 4.36 (1H, CH, C7), 7.21 (1H, CH, s, C8), 6.92 (1H, CH, d, *J* = 10.76 Hz, C11), 7.15 (1H, CH, d, *J* = 10.76 Hz, C12), 3.61 (3H, CH_3_, s, C15), 3.86 (3H, CH_3_, s, C16), 3.83 (3H, CH_3_, s, C17), 3.90 (3H, CH_3_, s, C18), 1.95 (3H, CH_3_, s, C14), 7.22 (1H, NH, d, *J* = 7.36 Hz); ^13^C NMR (75 MHz, CD_3_CN, TMS, 25 °C, ppm). (C1), (C1a), (C2), (C3), (C4), (C4a), (C5), (C6), (C7), (C7a), (C8), (C9), (C10), (C11), (C12), C12a), (C13), (C14), (C15), (C16), (C17), (C18). FT IR (KBr, cm^−1^): 3441, 3359, 3125, 2998, 2970, 2938, 1650 ν_(CO)_, 1610 ν_(CO)_, 1552, 1490, 1252, 1135, 1093. Anal. El. analysis (calcd., found for C_22_H_25_NO_6_·KCl·3H_2_O): C (50.00, 49.97), H (5.87, 5.83), N (2.65, 2.60).

Complex **2-LiCl**: Yield 97%, 108 mg; m.p. ≥ 200 °C, UV (MeOH, λ_max_, [cm^−1^]): 349, 244, ^1^H NMR (CD_3_CN, 300.075 MHz, 25 °C, ppm): 6.57 (1H, CH, s, C4), 2.53 (2H, CH_2_, C5), 1.96 (2H, CH_2_, C6), 4.66 (1H, CH, C7), 7.68 (1H, CH, s, C8), 7.31 (1H, CH, d, *J* = 11.3 Hz, C11), 7.55 (1H, CH, d, *J* = 11.3 Hz, C12), 3.63 (3H, CH_3_, s, C15), 3.95 (3H, CH_3_, s, C16), 3.91 (3H, CH_3_, s, C17), 2.02 (3H, CH_3_, s, C14), 6.93 (1H, NH, d); ^13^C NMR (75 MHz, CD_3_CN, TMS, 25 °C, ppm). 153.63 (C1), 125.94 (C1a), 141.67 (C2), 150.94 (C3), 107.33 (C4), 134.28 (C4a), 29.76 (C5), 37.59 (C6), 52.73 (C7), 151.18 (C7a), 119.37 (C8), 170.12 (C9), 169.75 (C10), 122.29 (C11), 141.37 (C12), 136.12 (C12a), 170.24 (C13), 22.94 (C14), 61.44 (C15), 61.38 (C16), 56.10 (C17). FT IR (KBr, cm^−1^): 3367, 3145, 2998, 2969, 2938, 2859, 1649 ν_(CO)_, 1610 ν_(CO)_, 1552, 1491, 1253, 1136, 1094. Anal. El. analysis (calcd., found for C_21_H_23_NO_6_·LiCl·3H_2_O): C (52.88, 52.82), H (6.08, 6.05), N (2.93, 2.92).

Complex **2-NaCl**: Yield 95%, 110 mg; m.p. = 162–164 °C, UV (MeOH, λ_max_, [cm^−1^]): 348, 243, ^1^H NMR (CD_3_CN, 300.075 MHz, 25 °C, ppm): 6.56 (1H, CH, s, C4), 2.53 (2H, CH_2_, C5), 1.95 (2H, CH_2_, C6), 4.67 (1H, CH, C7), 7.27 (1H, CH, s, C8), 7.33 (1H, CH, d, *J* = 11.6 Hz, C11), 7.60 (1H, CH, d, *J* = 11.6 Hz, C12), 3.63 (3H, CH_3_, s, C15), 3.94 (3H, CH_3_, s, C16), 3.91 (3H, CH_3_, s, C17), 2.03 (3H, CH_3_, s, C14), 6.79 (1H, NH, d); ^13^C NMR (75 MHz, CD_3_CN, TMS, 25 °C, ppm). (C1), (C1a), (C2), (C3), (C4), (C4a), (C5), (C6), (C7), (C7a), (C8), (C9), (C10), (C11), (C12), C12a), (C13), (C14), (C15), (C16), (C17). FT IR (KBr, cm^−1^): 3359, 3125, 2998, 2972, 2938, 2859, 1650 ν_(CO)_, 1610 ν_(CO)_, 1552, 1490, 1252, 1135, 1093. Anal. El. analysis (calcd., found for C_21_H_23_NO_6_·NaCl·H_2_O): C (54.54, 54.50), H (5.41, 5.37), N (3.03, 3.00).

Complex **2-KCl**: Yield 97%, 116 mg; m.p. = 160–162 °C, UV (MeOH, λ_max_, [cm^−1^]): 350, 244, ^1^H NMR (CD_3_CN, 300.075 MHz, 25 °C, ppm): 6.57 (1H, CH, s, C4), 2.52 (2H, CH_2_, C5), 1.96 (2H, CH_2_, C6), 4.67 (1H, CH, C7), 7.58 (1H, CH, s, C8), 7.34 (1H, CH, d, *J* = 11.5 Hz, C11), 7.60 (1H, CH, d, *J* = 11.5 Hz, C12), 3.64 (3H, CH_3_, s, C15), 3.95 (3H, CH_3_, s, C16), 3.91 (3H, CH_3_, s, C17), 2.02 (3H, CH_3_, s, C14), 7.12 (1H, NH, d); ^13^C NMR (75 MHz, CD_3_CN, TMS, 25 °C, ppm). (C1), (C1a), (C2), (C3), (C4), (C4a), (C5), (C6), (C7), (C7a), (C8), (C9), (C10), (C11), (C12), C12a), (C13), (C14), (C15), (C16), (C17). FT IR (KBr, cm^−1^): 3359, 3126, 2998, 2969, 2938, 2858, 1650 ν_(CO)_, 1610 ν_(CO)_, 1552. Anal. El. analysis (calcd., found for C_21_H_23_NO_6_KCl·H_2_O): C (52.72, 52.68), H (5.23, 5.19), N (2.93, 2.90).

Complex **3-LiCl**: Yield 93%, 106 mg; m.p. = 171–173 °C, UV (MeOH, λ_max_, [cm^−1^]): 370, 256, ^1^H NMR (CD_3_CN, 300.075 MHz, 25 °C, ppm): 6.80 (1H, CH, s, C4), 2.24 (2H, CH_2_, C5), 1.82 (2H, CH_2_, C6), 4.33 (1H, CH, C7), 7.04 (1H, CH, s, C8), 7.27 (1H, CH, d, *J* = 10.6 Hz, C11), 7.15 (1H, CH, d, *J* = 10.6 Hz, C12), 3.54 (3H, CH_3_, s, C15), 3.84 (3H, CH_3_, s, C16), 3.79 (3H, CH_3_, s, C17), 2.41 (3H, CH_3_, s, C18), 1.84 (3H, CH_3_, s, C14), 8.74 (1H, NH, d, *J* = 7.30 Hz); ^13^C NMR (75 MHz, CD_3_CN, TMS, 25 °C, ppm). 150.43 (C1), 125.35 (C1a), 140.75 (C2), 153.16 (C3), 107.84 (C4), 134.41 (C4a), 29.18 (C5), 35.65 (C6), 51.40 (C7), 151.18 (C7a), 127.89 (C8), 181.19 (C9), 157.30 (C10), 126.65 (C11), 134.08 (C12), 137.53 (C12a), 168.66 (C13), 22.46 (C14), 60.93 (C15), 60.73 (C16), 55.90 (C17), 14.40 (C18). FT IR (KBr, cm^−1^): 3417, 2941, 2856, 1636 ν_(CO)_, 1603 ν_(CO)_, 1539, 1487, 1236, 1135, 1093, 845. Anal. El. analysis (calcd., found for C_22_H_25_NO_5_SLiCl·1.5H_2_O): C (54.48, 54.44), H (5.78, 5.75), N (2.88, 2.84), S (6.60, 6.58).

Complex **3-NaCl**: Yield 95%, 110 mg; m.p. = 110–112 °C, UV (MeOH, λ_max_, [cm^−1^]): 370, 256, ^1^H NMR (CD_3_CN, 300.075 MHz, 25 °C, ppm): 6.79 (1H, CH, s, C4), 2.23 (2H, CH_2_, C5), 1.84 (2H, CH_2_, C6), 4.36 (1H, CH, C7), 7.02 (1H, CH, s, C8), 7.28 (1H, CH, d, *J* = 10.6 Hz, C11), 7.15 (1H, CH, d, *J* = 10.6 Hz, C12), 3.55 (3H, CH_3_, s, C15), 3.84 (3H, CH_3_, s, C16), 3.80 (3H, CH_3_, s, C17), 2.41 (3H, CH_3_, s, C18), 1.84 (3H, CH_3_, s, C14), 8.65 (1H, NH, d, *J* = 7.30 Hz); ^13^C NMR (75 MHz, CD_3_CN, TMS, 25 °C, ppm). 150.41 (C1), 125.31 (C1a), 140.72 (C2), 153.13 (C3), 107.80 (C4), 134.38 (C4a), 29.17 (C5), 35.66 (C6), 51.32 (C7), 151.13 (C7a), 127.83 (C8), 181.14 (C9), 157.28 (C10), 126.61 (C11), 134.06 (C12), 137.48 (C12a), 168.56 (C13), 22.45 (C14), 60.91 (C15), 60.71 (C16), 55.68 (C17), 14.37 (C18). FT IR (KBr, cm^−1^): 3277, 2934, 2836, 1659 ν_(CO)_, 1605 ν_(CO)_, 1541, 1485, 1235, 1136, 1094, 842. Anal. El. analysis (calcd., found for C_22_H_25_NO_5_SNaCl·2H_2_O): C (51.81, 51.78), H (5.69, 5.64), N (2.75, 2.71), S (6.28, 6.25).

Complex **3-KCl**: Yield 97%, 115 mg; m.p. = 147–149 °C; UV (MeOH, λ_max_, [cm^−1^]): 364, 235, ^1^H NMR (CD_3_CN, 300.075 MHz, 25 °C, ppm): 6.79 (1H, CH, s, C4), 2.23 (2H, CH_2_, C5), 1.83 (2H, CH_2_, C6), 4.33 (1H, CH, C7), 7.03 (1H, CH, s, C8), 7.27 (1H, CH, d, *J* = 10.6 Hz, C11), 7.15 (1H, CH, d, *J* = 10.6 Hz, C12), 3.53 (3H, CH_3_, s, C15), 3.87 (3H, CH_3_, s, C16), 3.77 (3H, CH_3_, s, C17), 2.41 (3H, CH_3_, s, C18), 1.81 (3H, CH_3_, s, C14), 8.63 (1H, NH, d, *J* = 7.30 Hz); ^13^C NMR (75 MHz, CD_3_CN, TMS, 25 °C, ppm). 150.41 (C1), 125.32 (C1a), 140.72 (C2), 153.14 (C3), 107.81 (C4), 134.39 (C4a), 29.17 (C5), 35.66 (C6), 51.33 (C7), 151.15 (C7a), 127.85 (C8), 181.15 (C9), 157.29 (C10), 126.62 (C11), 134.07 (C12), 137.49 (C12a), 60.92 (C13), 60.71 (C14), 55.86 (C15), 168.58 (C16), 22.45 (C17), 14.37 (C18). FT IR (KBr, cm^−1^): 3281, 2935, 2836, 1660 ν_(CO)_, 1605 ν_(CO)_, 1542, 1485, 1235, 1136, 1094, 843. Anal. El. analysis (calcd., found for C_22_H_25_NO_5_S·KCl·H_2_O): C (51.91, 51.92), H (5.30, 5.34), N (2.70, 2.68), S (6.29, 6.27).

#### 3.1.2. Measurements

The NMR spectra of colchicine **1**, colchiceine **2**, and 10-methylthiocolchicine **3**, as well as their 1:1 chloride salt complexes (0.07 mol L^−1^) with monovalent metal cations, were recorded in CD_3_CN solutions using a Varian Gemini 300 MHz spectrometer [21,22,23,24,25,26,27]. All spectra were locked to the CD_3_CN deuterium resonance.

^1^H NMR measurements in CD_3_CN were performed at an operating frequency of 300.075 MHz; rotation angle, pw = 45°; spectrum width, sw = 4500 Hz; acquisition time, at = 2.0 s; relaxation delay, d1 = 1.0 s; T = 293.0 K; and using TMS as the internal standard. No windowing or zero filling was applied. The digital resolution was 0.2 Hz per point. The chemical shift error was 0.01 ppm. The ^13^C NMR spectra were recorded at an operating frequency of 75.454 MHz; pw = 60°; sw = 19,000 Hz; at = 1.8 s; d1 = 1.0 s; T = 293.0 K; and TMS as the internal standard. The line broadening parameters were 0.5 or 1 Hz. The chemical shift error was 0.01 ppm. The ^1^H and ^13^C NMR signals were assigned for each species using one- or two-dimensional spectra (COSY, HETCOR, and HMBC).

FT IR spectra of colchicine and its 1:1 complex (0.07 mol dm^−3^) were recorded in the mid-infrared range in KBr pellets for colchicine **1**, colchicine **2**, 10-methylthiocolchicine **3**, and their complexes with lithium, sodium, and potassium chlorides. The spectra were recorded using an FT IR IFS 113v spectrophotometer (Bruker, Karlsruhe) equipped with a DTGS detector; resolution 2 cm^−1^, NSS = 125. The Happ-Genzel apodization function was used [21,22,23,24,25,26,27]. To avoid interference, a cell with Si windows and wedge-shaped layers (average layer thickness: 170 μm) was used. The substances were always stored in a carefully dried and CO2-free glove box.

UV–Vis spectra were recorded in methanol using a JASCO V-650 spectrophotometer in the measurement range of 200–600 nm. ESI MS (electrospray ionization) mass spectra were recorded on a Waters/Micromass (Manchester, UK) ZQ mass spectrometer equipped with a Harvard Apparatus syringe pump. All samples were prepared at CD_3_CN.

Measurements were performed for two types of samples: solutions of colchicine or colchiceine or 10-methylthiocolchicine (5 × 10^−5^ mol dm^−3^) with each of the cations Li^+^, Na^+^, and K^+^ (2.5 × 10^−4^ mol dm^−3^) taken separately, and cations Li^+^, Na^+^, and K^+^ (5 × 10^5^/mol dm^−3^) taken together. The samples were introduced into the ESI source using a Harvard pump at a flow rate of 20 μL min^−1^. The ESI MS source potentials were as follows: capillary 3 kV, lens 0.5 kV, and extractor 4 V. Standard ESI MS mass spectra were recorded at a voltage of 30 V. The source temperature was 120 °C and the desolvation temperature was 300 °C. Nitrogen was used as the nebulizing and desolvation gas at flow rates of 100 and 300 dm^3^ h^−1^, respectively. Mass spectra were obtained in positive ion detection mode with a mass resolution of 1 *m*/*z*. The mass range for the ESI MS experiments was from *m*/*z* = 100 to *m*/*z* = 1500. The elemental analysis of colchicine **1**, colchiceine **2**, and 10-methylthiocolchicine **3** complexes was performed using a Vario ELIII device (Elementar, Rhein-Main-Gebiet, Germany).

### 3.2. Biological Tests

#### 3.2.1. Fungicidal Activity

Fungal strains. The following mitosporous fungi were used as test organisms: *Aspergillus niger* van Tiegen ATCC 6275, *Aspergillus versicolor* ATCC 11730, *Paecilomyces variotii* ATCC 18502, *Penicillium funiculosum* ATCC 11797, *Chaetomium globosum* ATCC 6205, *Aureobasidium pullulans* ATCC 9348, *Penicillium cyclopium* Westling, and *Trichoderma viride Persoon ex* S.F. Gray aggr. The fungal strains were obtained from the collection of the Federal Institute for Materials Research and Testing BAM or the Institute of Wood Chemistry Technology (Poznań University of Life Sciences) [23,25,26,27,30,31,32,35].

Microdilution broth method. The antifungal activity of the new complexes of colchicine **1**, colchicine *2*, and 10-methylthio-colchicine **3** was evaluated using a microdilution broth test in a 96-well microdilution plate. The MIC value was defined as the lowest concentration of the antifungal agent at which the solution was optically clear. The 96-well plates were prepared by adding 100 μL of double-concentrated potato dextrose agar (Sigma-Aldrich, Darmstadt, Germany) as a culture medium to each well.

Next, 100 μL of serial dilutions were transferred to subsequent wells. Ten µL of freshly prepared fungal spore suspension (10^−5^ to 10^−6^ CFU/mL) was added to each well using a micropipette. The fungal spore suspensions were obtained from two-week-old agar media. The results obtained for each compound were compared with control wells using a commercially available fungicide such as 3-iodo-2-propynyl butylcarbamate (IPBC) (Preventol^®^ MP100 from Lanxess) and chalcone [39] (Sigma-Aldrich, Germany) as a molecule with broad biological activity. The last well contained 200 μL of PDA with a fungal suspension to confirm cell viability (viability control) [40].

The plates were processed by mixing on a plate shaker (300 rpm) for 30 s and incubated aerobically for 3–5 days in a humid chamber with relative humidity (RH) above 95% and a temperature of 28 ± 1 °C in the dark. To verify the repeatability and accuracy of the method using microtiter plates, each compound was tested three times.

#### 3.2.2. Cytotoxic Activity

The cytotoxic activity of the synthesized colchicine analogues was evaluated using the SKOV-3 ovarian cancer cell line. This cell line was obtained from the European Collection of Cell Cultures via Sigma-Aldrich Poland. All other chemicals used in the study were also purchased from Sigma-Aldrich. Cells were seeded in 96-well plates at a density of 15,000 cells per well and incubated at 37 °C in a humidified atmosphere containing 5% CO_2_. The culture medium consisted of DMEM supplemented with 2 mM glutamine, 100 µg/mL streptomycin, 100 U/mL penicillin, and 10% fetal bovine serum (FBS). Following overnight attachment, the test compounds—dissolved in DMSO—were added to the wells and incubated for 72 h. Control wells were treated with DMSO alone, with the final DMSO concentration maintained at 0.1% in both treated and control groups. Cell viability was assessed using the MTT assay, which quantifies metabolically active cells based on their ability to reduce the yellow dye 3-(4,5-dimethylthiazol-2-yl)-2,5-diphenyl tetrazolium bromide (MTT) to a purple formazan product [41]. Since formazan accumulates only in viable cells, the resulting colorimetric signal correlates with the number of living cells in the sample. At the end of the incubation period, the plates were centrifuged, and the culture medium was replaced with 200 µL of fresh medium containing 0.5 mg/mL MTT. After a 3 h incubation, the resulting formazan crystals were dissolved in 150 µL of DMSO, and absorbance was measured at 550 nm using a microplate reader (BioTek Elx-800, BioTek Instruments, Inc., Winooski, VT, USA). Cytotoxicity was expressed as the percentage of absorbance relative to untreated control cells. The half-maximal inhibitory concentration (IC_50_) values were determined using GraphPad Prism version 8.4.3 (GraphPad Software, San Diego, CA, USA).

### 3.3. In Silico Screening/Calculations

#### 3.3.1. Molecular Lipophilicity

Web tools were used to predict parameters such as water solubility, lipophilicity, toxicity, LD_50_ values, and other factors and physicochemical properties for all 12 tested compounds. MolInspiration Cheminformatics tool was used with ALOGPS 2.1. Virtual Computational Chemistry Laboratory was used for LogP calculations (data given in Table 6), and the MolInspiration Galaxy 3D generator (data given in Figure S25) and Galaxy Visualizer were used to visualize the molecular lipophilicity potential (MLP) on the molecular surface.

The SwissADME web tool was also used to predict the lipophilicity LogP values (by five models) of the 12 compounds chosen for biological tests and to predict water solubility with LogS values. The data are summarized in Table 5 and Table 6 (see Supplementary Information **5.** Physicochemical properties prediction [42,43,44,45,46,47,48,49,50,51,52,53,54]).

The ProTox-II platform is divided into five different classification steps: 1. acute toxicity (oral toxicity model with six different toxicity classes 1–6); 2. organ toxicity (one model—hepatotoxicity); 3. toxicological endpoints (four models); 4. toxicological pathways (12 models); and 5. toxicity targets (15 models). Platform conducts predictions on LD_50_ values. Platform used Tox21 (the “Toxicology in the 21st century” program, a federal collaboration involving NIH, the Environmental Protection Agency, and the Food and Drug Administration, which aims to develop better toxicity assessment methods).

#### 3.3.2. DFT Theoretical Studies

All structures needed for theoretical calculations were obtained based on the known crystal structure of colchicine dehydrate 1 [55]. Energy calculations were performed within the DFT framework at the M06/6-31+g(d,p) level of theory [56,57,58,59,60,61,62], selected based on the results of extensive comparative studies in [7] and recommended for calculations of compounds containing metal atoms [21,24,37,63,64]. Partial atomic charges were calculated at the same level of theory. In our studies, we selected Mulliken [65] point charges. Counterpoise correction [66,67] was calculated to assess the Basis Set Superposition Error (BSSE). To access the impact of solvent on the results, calculations were performed in the presence of solvent using the PCM model [68]. We used the same solvent as for complex synthesis (methanol). All quantum mechanics calculations were performed with GAUSSIAN 09 [69].

#### 3.3.3. Molecular Modeling

The initial 3-dimensional structures of the studied complexes were prepared, based on known colchicine-site inhibitor geometries and previous studies on related alkali metal complexes, by the manual superposition of **3-Li** geometry (conformers A, B, and C obtained by DFT calculations) to the colchicine-binding site in the crystal structure of human α/β-tubulin heterodimer (PDB ID: 5EYP) [70]. Throughout the manuscript, the term “tubulin” refers to the α/β heterodimer, unless explicitly stated otherwise. Although 2:1 metal-ligand complexes were identified as energetically favorable species in solution-phase DFT calculations, QM/MM simulations were restricted to the 1:1 complexes. This choice reflects the steric limitations of the colchicine-binding pocket and the experimentally observed predominance of the 1:1 stoichiometry under biologically relevant conditions. Subsequently, full optimization of the complexes was performed using the QM/MM: ONIOM protocol, implemented in the GAUSSIAN 16 software [71]. Although exhaustive conformational sampling was not performed, full QM/MM optimization allowed significant rearrangements of both the ligand and Li^+^ coordination, ensuring that the final geometries were not artificially constrained to the initial pose. The ONIOM calculations were intended to compare representative, biologically relevant binding modes rather than to explore the entire conformational landscape.

The remaining parts of the receptor were assigned to the MM region, described by the AMBER [72] force field. Optimization of the binding site was performed using the DFT B3LYP method [73,74] in combination with the split-valence basis set 6-31G* [75,76,77,78,79].

To study the importance and nature of ligand-protein (L–P) interactions, single-point FMO-EDA [80] calculations were performed at the MP2/6–31G* level using the GAMESS program [81]. While MP2 results are basis-set-dependent and larger basis sets could provide more rigorous absolute energies, the chosen level represents a computationally feasible compromise. The FMO calculations were performed for the ligand and β-tubulin binding site. Pair interaction energies (PIEs) and all contributions to total energies (electrostatic: Ees, dispersion: Edis, charge-transfer: Ect, exchange repulsion: Eex, and Gibbs solvation energy: ΔGsolv) were calculated as previously described [80,82,83,84,85,86,87]. Gibbs solvation energy was calculated based on the PCM model. GaussView 5.0 [88] and FACIO [89] were used to prepare the systems and analyze the results.

#### 3.3.4. In Silico Screening

A KNIME workflow was used to automate the virtual screening of compounds, applying a predefined, indication-specific, knowledge-based set of property threshold values. These included logP (n-octanol-water partition coefficient), logS (natural logarithm (base 10) of solubility measured in moles/liter), molecular weight, acceptors and donors, and proprietary BASF filters. Based on these filters, compounds were either rejected or assigned to the appropriate indications for testing in BASF’s preliminary screening [90].

## 4. Conclusions

In summary, the complexation of colchicine 1, colchiceine 2, and 10-methylthio-colchicine 3 with alkali metal chlorides such as Li^+^, Na^+^, and K^+^ resulted in selective and compound-dependent changes in biological activity rather than a uniform enhancement.

The cytotoxic activity of the tested complexes **3-LiCl**, **3-NaCl**, and **3-KCl** against the SKOV-3 cancer cell line confirmed that these compounds exhibited activity comparable to that of 10-methylthiocolchicine and were significantly more active than the commonly used doxorubicin. In contrast, the antifungal activity of the tested compounds was limited to a narrow subgroup of microfungal species. The 10-methylthiocolchicine complexes showed only some activity against *P. cyclopium* and *A. pullulans*, while the **2-LiCl**, **2-NaCl**, and **2-KCl** complexes were the most active, particularly against *A. pullulans* and *Ch. globosum*. This species-specific activity suggests a rather limited, niche antifungal potential rather than a broad spectrum of activity. Cytotoxic and antifungal activity is related to the water solubility and lipophilicity of the tested compounds, which determine the interactions with lipids, penetration through cell membranes, and binding to proteins. Based on the analyses of the water solubility and lipophilicity of the tested compounds, it was found that some of them have optimal bioavailability, while the others have fairly good or satisfactory bioavailability. The LogP value obtained using the consensus method (which involves averaging the results of several validated methods) provided the best overall accuracy, indicating that the majority of the compounds studied exhibit a potentially optimal bioavailability and that their LogP values fall within the range defined by Lipinski’s rule.

Quantum chemical calculations showed that the most energetically favorable complexes of 10-methylthiocolchicine with the lithium cation were obtained when one or both 10-methylthio-colchicine molecules were coordinated via the oxygen atoms O4 and O5. Furthermore, our calculations showed that, among all the complexes studied with stoichiometry 1:1 and 2:1, the most energetically favorable interaction was Li+…O4. The calculations confirmed that 10-methylthiocolchicine coordinated with lithium strongly stabilizes the active complex of the ligand and protein, which may contribute to the observed cytotoxic activity.

## Figures and Tables

**Table 1:** Main peaks in the ESI ES^+^ mass spectra of the complexes measured at cv = 30 V and relative intensities [%]; see SI. **L** = **1** colchicine, **2** colchiceine, **3** 10-methylthiocolchicine.

Cpd.	1:1 Complex L-M^+^[%]	2:1 Complex 2 L-M^+^[%]	3:1 Complex 3 L-M^+^[%]	1:2:1 Complex L-2M^+^-X^−^[%]	2:2:1 Complex 2 L-2M^+^-X^−^[%]
**1-LiCl**	406 [80]	805 [100]	1204 [10]	448 [10]	847 [8]
**1-NaCl**	422 [100]	821 [70]	-	480 [10]	-
**1-KCl**	438 [100]	837 [30]	-	512 [10]	-
**2-LiCl**	392 [100]	777 [33]	-	435 [10]	-
**2-NaCl**	408 [100]	794 [10]	-	-	-
**2-KCl**	424 [100]	809 [10]	-	-	-
**3-LiCl**	422 [100]	837 [15]	-	464 [65]	879 [20]
**3-NaCl**	438 [100]	853 [20]	-	496 [15]	-
**3-KCl**	454 [100]	869 [20]	-	528 [10]	-

**Table 2:** Calculated interaction energy for the studied interaction schemes of **3-Li^+^** complexes in vacuum and methanol obtained at the M06/6—31+g(d,p) level of theory.

Vacuum			Methanol
Complex	Interaction Energy (Raw) [kcal/mol]	Interaction Energy (Corrected) [kcal/mol]	Interaction Energy [kcal/mol]
1:1 Type A	−89.6	−88.8	−8.5
1:1 Type B	−62.2	−61.6	−2.5
1:1 Type C	−96.9	−96.6	−9.8
2:1 Type D	−135.2	−131.9	−25.4
2:1 Type E	−143.2	−139.8	−25.7

**Table 3:** The results of bioassays for minimal inhibitory concentration (MIC) [μg/mL and [mMol/mL]] against eight microfungi.

Complex	MIC [μg/mL and [mMol/mL]] of Different Fungal Species							
	*A. niger*	*A. versicolor*	*P. variotti*	*P. funiculosum*	*T. viride*	*P. cyclopium*	*A. pullulans*	*Ch. globosum*
**1**	>4000	>4000	>4000	>4000	>4000	>4000	1 ± 0.0 [2.5 × 10^−10^]	>4000
**1-LiCl**	>4000	>4000	>4000	>4000	>4000	>4000	>4000	>4000
**1-NaCl**	>4000	>4000	>4000	>4000	>4000	>4000	>4000	>4000
**1-KCl**	>4000	>4000	>4000	>4000	>4000	>4000	>4000	>4000
**2**	500 ± 0.0 [1.3 × 10^−10^]	500 ± 0.0 [1.3 × 10^−10^]	260 ± 0.0 [6.8 × 10^−11^]	1000 ± 0.0 [2.6 × 10^−10^]	500 ± 0.0 [1.3 × 10^−10^]	500 ± 0.0 [1.3 × 10^−10^]	2 ± 0.0 [5.5 × 10^−12^]	2000 ± 0.0 [5.2 × 10^−10^]
**2-LiCl**	>4000	>4000	>4000	1000 ± 0.0 [2.3 × 10^−10^]	>4000	>4000	130 ± 0.0 [3.0 × 10^−11^]	65 ± 0.0 [1.5 × 10^−11^ ]
**2-NaCl**	>4000	>4000	>4000	1000 ± 0.0 [2.2 × 10^−10^]	>4000	>4000	260 ± 0.0 [5.8 × 10^−11^]	65 ± 0.0 [1.5 × 10^−11^]
**2-KCl**	>4000	>4000	>4000	1000 ± 0.0 [2.2 × 10^−10^]	>4000	>4000	500 ± 0.0 [1.1 × 10^−10^]	65 ± 0.0 [1.4 × 10^−11^]
**3**	>4000	>4000	>4000	>4000	>4000	>4000	>4000	>4000
**3-LiCl**	>4000	>4000	>4000	>4000	>4000	2000 ± 0.0 [4.4 × 10^−10^]	2000 ± 600 [4.4 × 10^−10^]	>4000
**3-NaCl**	>4000	>4000	>4000	>4000	>4000	2000 ± 0.0 [4.2 × 10^−10^]	2000 ± 600 [4.2 × 10^−10^]	>4000
**3-KCl**	>4000	>4000	>4000	>4000	>4000	2000 ± 0.0 [4.1 × 10^−10^]	2000 ± 0.0 [4.1 × 10^−10^]	>4000
**Chalcone** (fungicide) *	65 ± 0.0 [3.1 × 10^−11^]	2000 ± 0.0 [9.6 × 10^−10^]	1000 ± 0.0 [4.8 × 10^−10^]	130 ± 0.0 [6.2 × 10^−11^]	500 ± 0.0 [2.4 × 10^−10^]	260 ± 0.0 [1.2 × 10^−10^]	500 ± 0.0 [2.4 × 10^−10^]	130 ± 0.0 [6.2 × 10^−11^]
**IPBC** (fungicide) *	2 ± 0.0 [7 × 10^−12^]	2 ± 0.0 [7 × 10^−12^]	2 ± 0.0 [7 × 10^−11^]	2 ± 0.0 [7 × 10^−12^]	100 ± 0.0 [3.5 × 10^−8^]	2 ± 0.0 [7 × 10^−12^]	1 ± 0.0 [3.5 × 10^−12^]	5 ± 0.0 [1.7 × 10^−11^]

* commonly used fungicides.

**Table 4:** IC_50_ values of the compounds tested, **1**, **2**, **3**, and chloride complexes against the SKOV-3 ovarian cell line after 72 h incubation. Data were obtained from triplicate experiments, presented as means ± SD. Colchicine **1** and doxorubicin were used as positive control.

**Compounds**	**IC_50_ [nM/mL]**	** 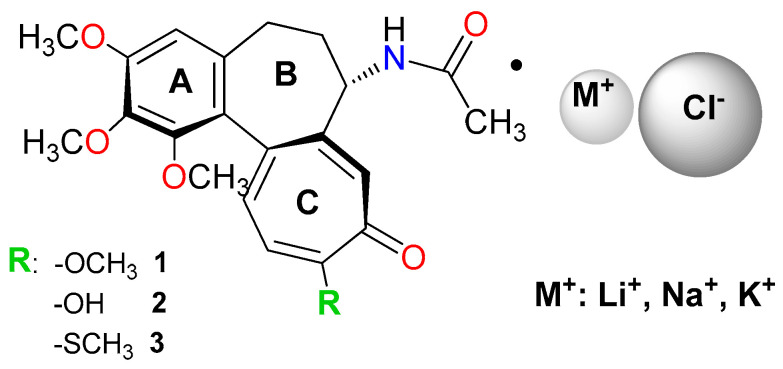 **
**1**	36.50 ± 5.01	
**1-LiCl**	26.17 ± 3.09	
**1-NaCl**	24.90 ± 1.55	
**1-KCl**	29.05 ± 0.87	
**2**	334.73 ± 3.38	
**2-LiCl**	372.80 ± 8.00	
**2-NaCl**	150.61 ± 2.18	
**2-KCl**	72.45 ± 1.64	
**3**	5.80 ± 0.49	
**3-LiCl**	3.36 ± 0.16	
**3-NaCl**	4.28 ± 0.17	
**3-KCl**	3.24 ± 0.43	
**doxorubicin**	3265.89 ± 33.24.17	

**Table 5:** Water solubility of tested compounds experimental [mg/mL] and predicted by the SwissADME web tool.

Compound	Water Solubility			
	LogS ESOL/Class	LogS Ali/Class	LogS SILICOS-IT/Class	[mg/mL]
**1**	−2.90/ soluble	−2.36/ soluble	−6.66/ poorly soluble	45
**1-LiCl**	−3.94/ soluble	−3.84/ soluble	−5.97/ moderately soluble	40
**1-NaCl**	−4.04/ moderately soluble	−3.84/ soluble	−6.01/ poorly soluble	38
**1-KCl**	−4.14/ moderately soluble	−3.84/ soluble	−6.05/ poorly soluble	40
**2**	−3.00/ soluble	−2.77/ soluble	−5.97/ moderately soluble	0.125
**2-LiCl**	−4.03/ moderately soluble	−4.14/ moderately soluble	−5.29/ moderately soluble	0.2
**2-NaCl**	−4.14/ moderately soluble	−4.13/ moderately soluble	−5.32/ moderately soluble	0.1
**2-KCl**	−4.23/ moderately soluble	−4.14/ moderately soluble	−5.36/ moderately soluble	0.15
**3**	−3.46/ soluble	−3.46/ soluble	−7.01/ poorly soluble	7
**3-LiCl**	−4.57/ moderately soluble	−5.06/ moderately soluble	−6.01/ poorly soluble	8
**3-NaCl**	−4.67/ moderately soluble	−5.06/ moderately soluble	−6.05/ poorly soluble	7
**3-KCl**	−4.77/ moderately soluble	−5.06/ moderately soluble	−6.09/ poorly soluble	6
**doxorubicin**	−3.91/soluble	−5.20/ moderately soluble	−3.46/ soluble	50
**IPBC**	−2.52/soluble	−2.55/soluble	−2.66/soluble	insoluble
**chalcone**	−3.43/ soluble	−3.11/ soluble	−4.96/ moderately soluble	0.16

**Table 6:** Lipophilicity of 12 tested compounds and standard compounds predicted by web tools.

Comp.	Lipophilicity							
	LogP Mol Inspiration	SwissADME						Log P Protox II
		LogP (iLOGP)	LogP (XLOGP3)	LogP (WLOGP)	Log P (MLOGP)	Log P (SILICOS-IT)	Consensus Log P	
**1**	1.10	3.28	1.03	2.55	1.02	3.90	2.36	3.26
**1-LiCl**	1.10	0.00	2.45	3.28	1.00	0.18	1.38	3.79
**1-NaCl**	1.10	0.00	2.45	3.28	1.00	0.28	1.40	3.79
**1-KCl**	1.10	0.00	2.45	3.28	1.00	0.38	1.42	3.79
**2**	0.83	3.10	1.20	2.24	0.80	3.35	2.14	2.96
**2-LiCl**	0.83	0.00	2.74	3.11	0.79	0.78	1.48	2.86
**2-NaCl**	0.83	0.00	2.74	3.11	0.79	0.88	1.50	2.86
**2-KCl**	0.83	0.00	2.74	3.11	0.79	0.98	1.53	2.86
**3**	2.17	3.32	1.76	3.26	1.82	4.46	2.92	3.98
**3-LiCl**	2.17	0.00	3.30	3.99	1.80	0.28	1.87	4.50
**3-NaCl**	2.17	0.00	3.30	3.99	1.80	0.38	1.89	4.01
**3-KCl**	2.17	0.00	3.30	3.99	1.80	0.48	1.91	4.01
**doxorubicin**	0.57	2.16	1.27	−0.32	−2.10	1.17	0.44	0.70
**IPBC**	2.23	2.87	2.11	1.99	2.00	1.82	2.16	2.26
**chalcone**	3.81	2.54	3.08	3.47	3.44	3.95	3.30	3.58

## Data Availability

All data generated or analyzed during this study are included in this published article and in the Supplementary Information files.

## Supplementary Materials

The following supporting information can be downloaded at: https://www.mdpi.com/article/10.3390/ijms27072985/s1.
