# Supplementary material for: Bioactivity of Novel Colchicine, Colchiceine, and 10-Methylthiocolchicine Complexes with Lithium, Sodium, and Potassium Chlorides: Experimental and Theoretical Studies
Source: Int J Mol Sci. 2026 Mar 25;27(7):2985. doi: 10.3390/ijms27072985 (PMC13072876; doi:10.3390/ijms27072985)

Supplementary Information

Bioactivity of Novel Colchicine, Colchiceine, and 10-Methylthiocolchicine Complexes with Lithium, Sodium, and Potassium Chlorides: Experimental and Theoretical Studies

Joanna Kurek [1], Patrycja Kwaśniewska-Sip [2], Wojciech Jankowski [3], Krzysztof Myszkowski [4], Grzegorz Cofta [5], Marcin Hoffmann [3], Marek Murias [4], Rafał Kurczab [6] and Paweł Śliwa [7]

1. NMR of complexes
1.1. ^{1}H NMR and ^{13}C NMR of **1-LiCl Figs. S1-S2.** **2**
1.2. ^{1}H NMR and ^{13}C NMR of **1-NaCl Figs. S3-S4.** **3**
1.3. ^{1}H NMR and ^{13}C NMR of **1-KCl Figs. S5-S6.** **4**
1.4. ^{1}H NMR and ^{13}C NMR of **2-LiCl Figs. S7-S8.** **5**
1.5. ^{1}H NMR and ^{13}C NMR of **2-NaCl Figs. S9-S10.** **6**
1.6. ^{1}H NMR and ^{13}C NMR of **2-KCl Figs. S11-S12.** **7**
1.7. ^{1}H NMR and ^{13}C NMR of **3-LiCl Figs. S13-S14.** **8**
1.8. ^{1}H NMR and ^{13}C NMR of **3-NaCl Figs. S15-S16.** **9**
1.9. ^{1}H NMR and ^{13}C NMR of **3-KCl Figs. S17-S18.** **10**
2. FT IR spectra of complexes **Figs S19-S21.** **11**
3. ESI MS mass spectra **Figs. S22-24** **13**
4. Fungcidal activity of complexes **16**
4.1. Bioassay tests **Table S1.** **16**
4.2. The results of bioassay tests for minimal fungicidal concentration against eight microfungi species **Table S2.** **17**
5. Physicochemical properties predictions **18**
5.1. Physicochemical and ADME data of synthesized compounds and standard reference compounds as predicted by SwissADME web tool **Table S3.** **19**
5.2. Oral toxicity prediction results obtained by Protox II tool **Table S4.** **20**
6. Lipophilicity of compounds **Fig. S25** **21**
7. DFT calculations **Table S5-S9.** **27**
7.1. Optimized structures **F-H** with 3:1 stoichiometry **Fig S26** **28**
7.2. Optimized structures : **A-C** with 1:1 stoichiometry, **D** and **E** with 2:1 stoichiometry, F-H with 3:1 stoichiometry **Fig. S27** **29**
8. Impact of water solubility on the cytotoxic activity of tested compounds **Figure S28**. . **30**
9. Cytotoxic effects of tested compounds and their corresponding salts (LiCl, NaCl, KCl) on SKOV-3 cells assessed using the MTT assay **Fig. S29.** **31**

1. NMR spectra of complexes

1.1. Figure S1. ^{13}C NMR of **1-LiCl**

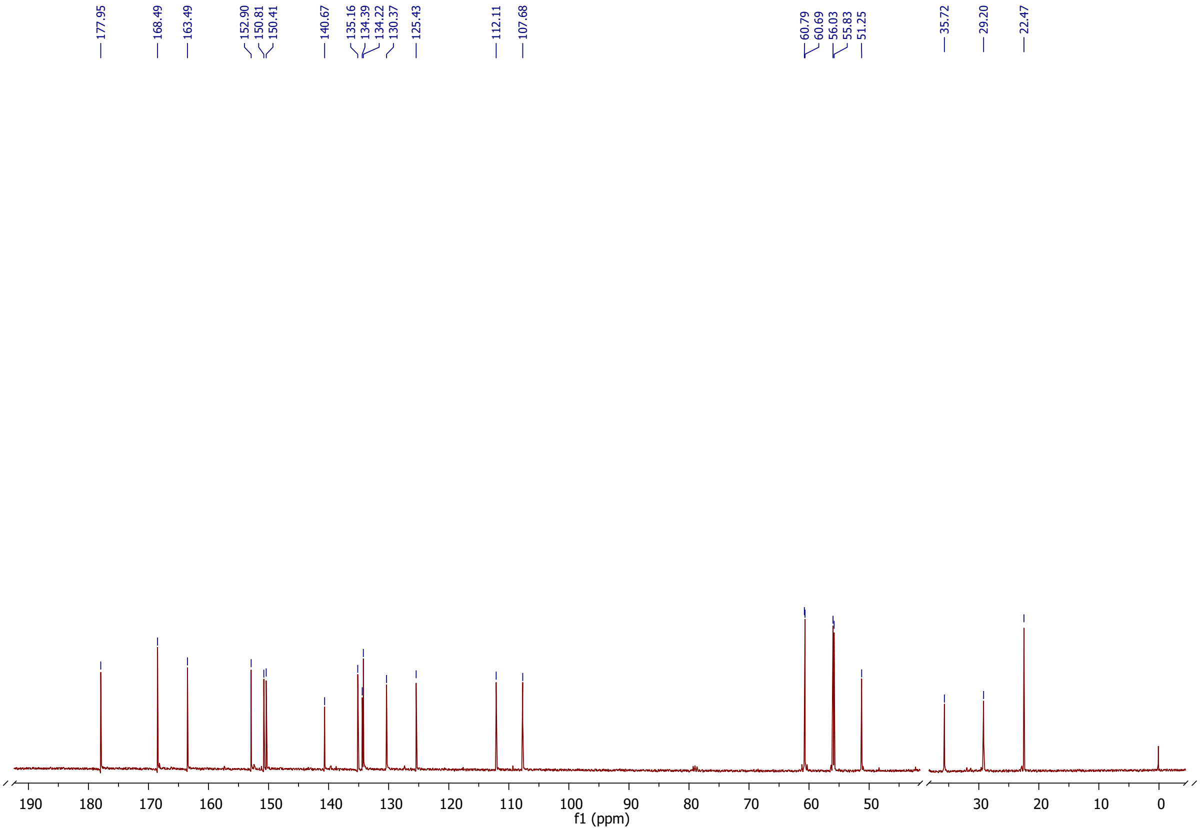

Figure S2. ^{1}H NMR of **1-LiCl**

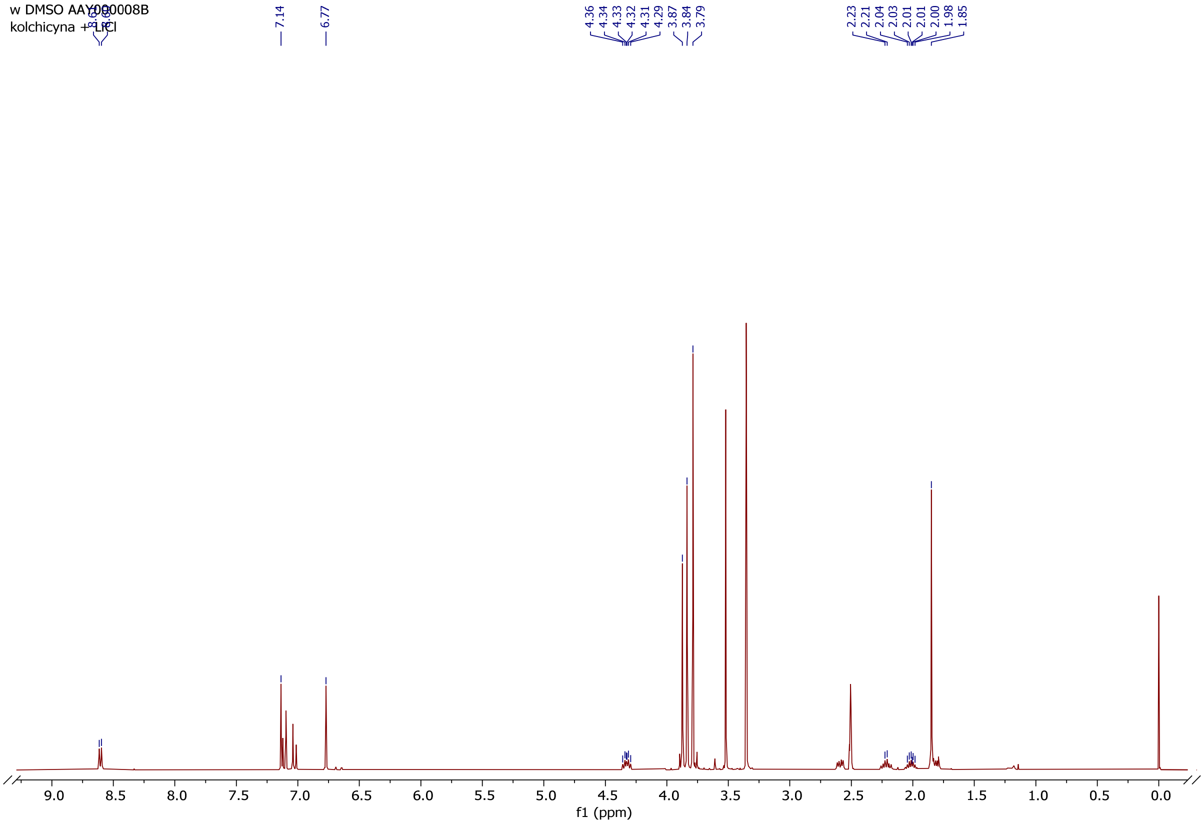

1.2. Figure S3. ^{13}C NMR of 1-NaCl

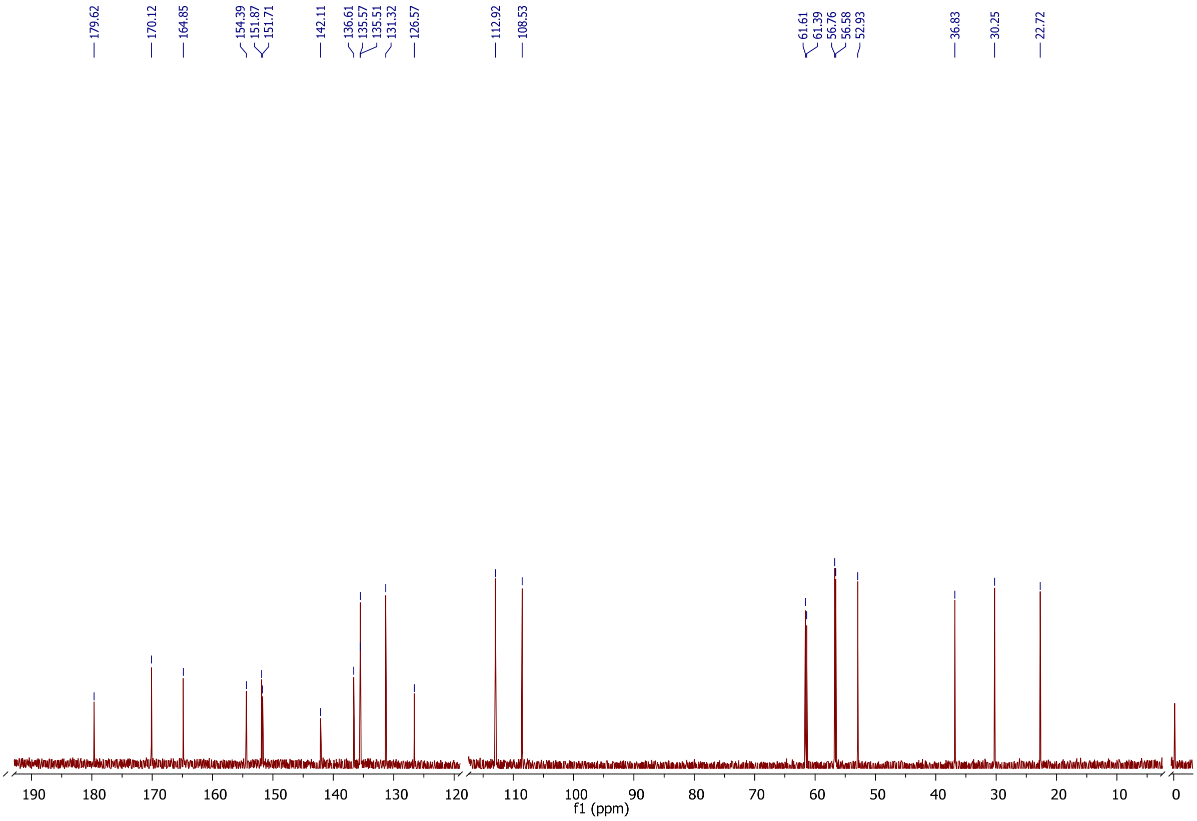

Figure S4. ^{1}H NMR of 1-NaCl

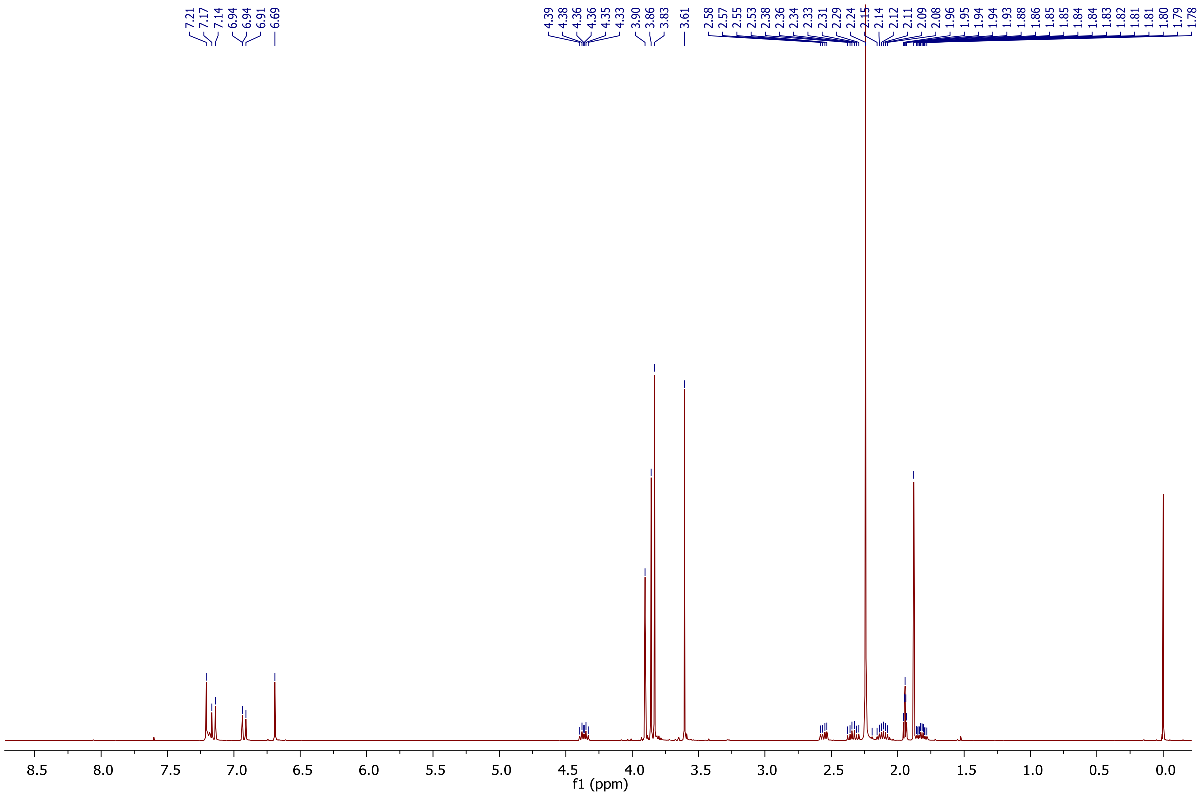

1.3. Figure S5. ^{13}C NMR of **1-KCl**

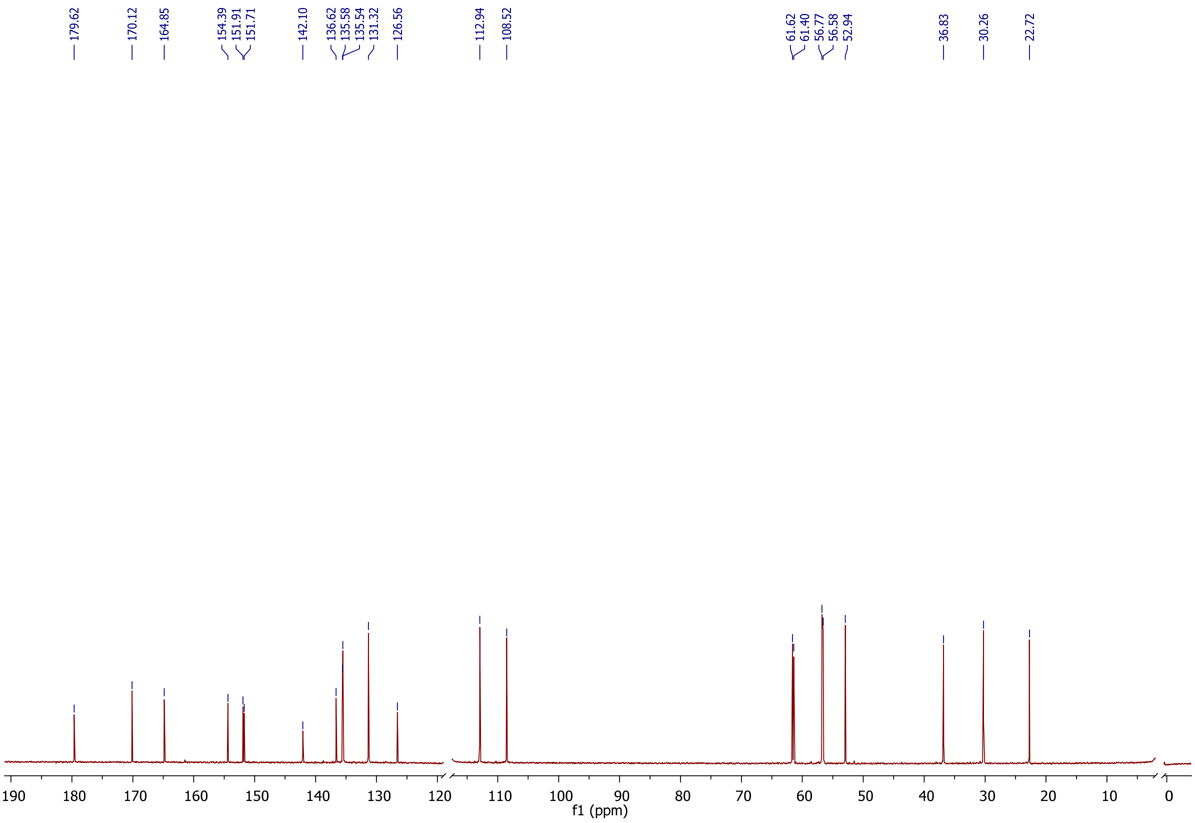

Figure S6. ^{1}H NMR of **1-KCl**

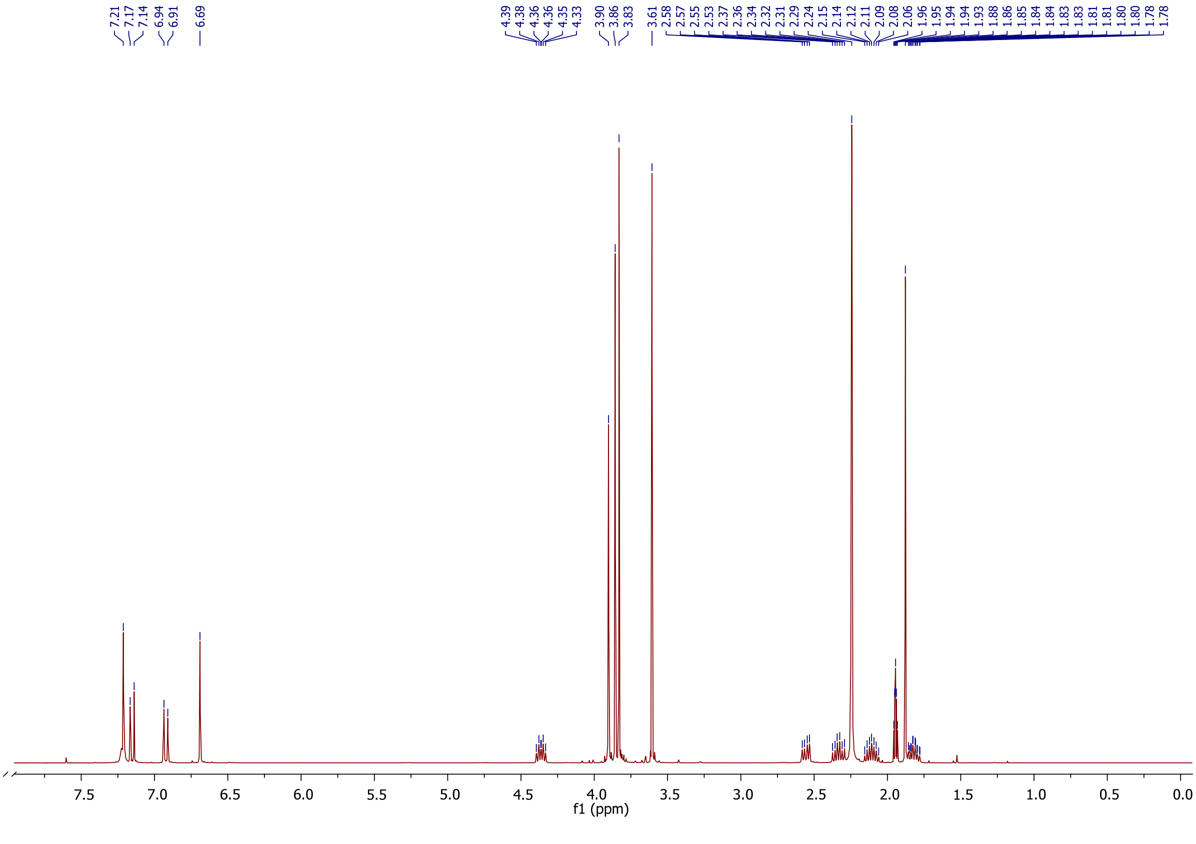

1.4. Figure S7. ^{13}C NMR of **2-LiCl**

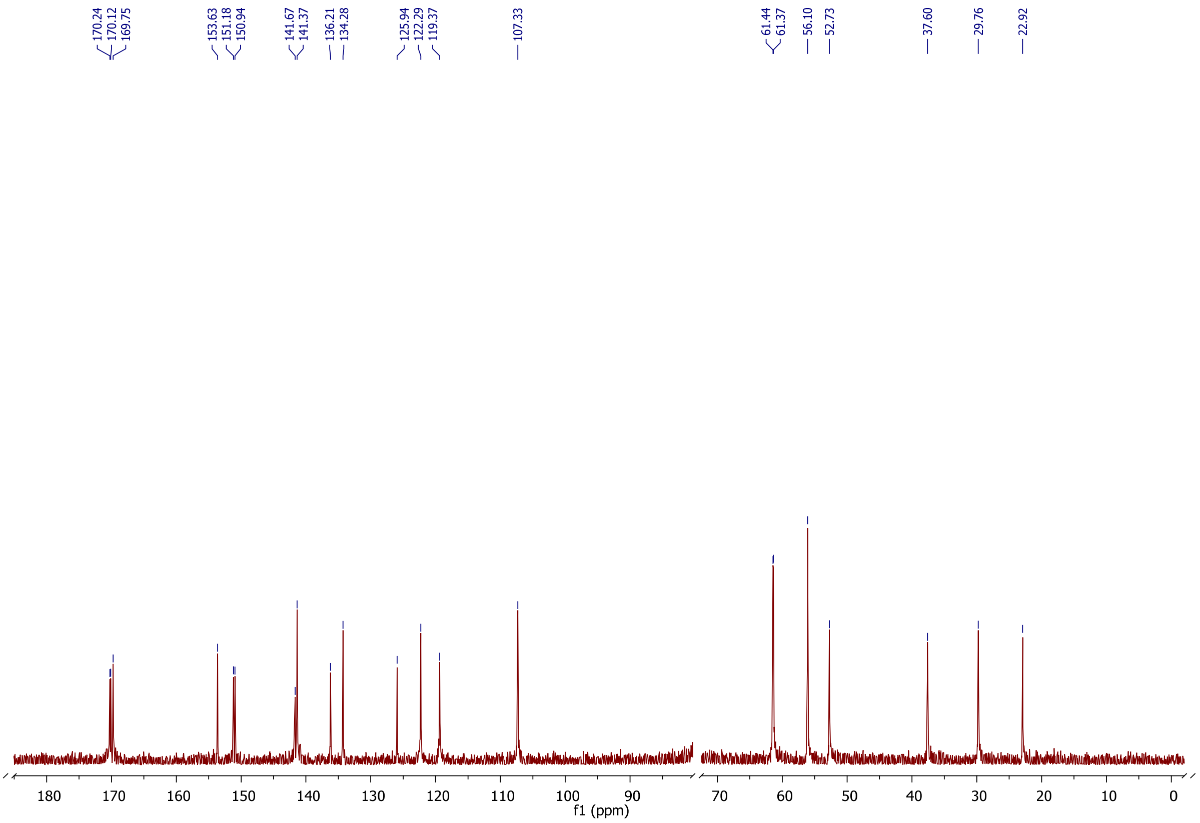

Figure S8. ^{1}H NMR of **2-LiCl**

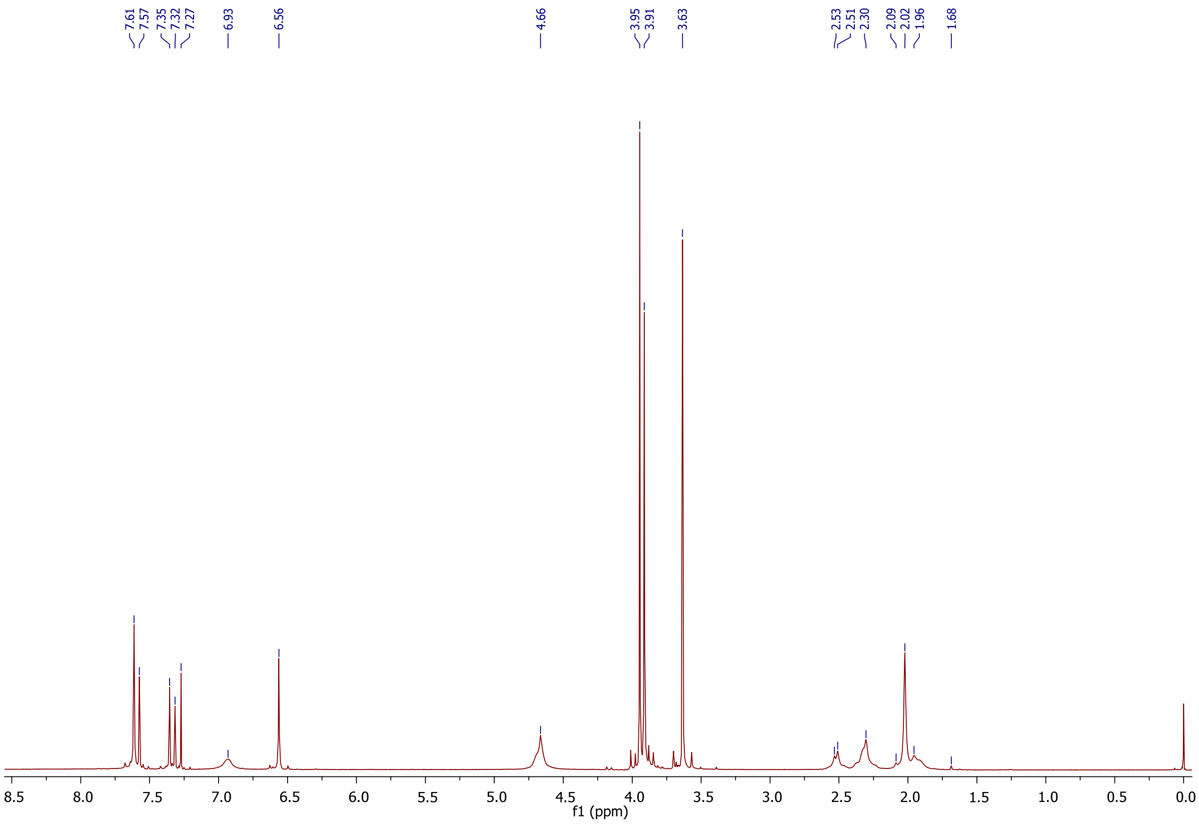

1.5. **Figure S9.** ^{13}C NMR of **2-NaCl**

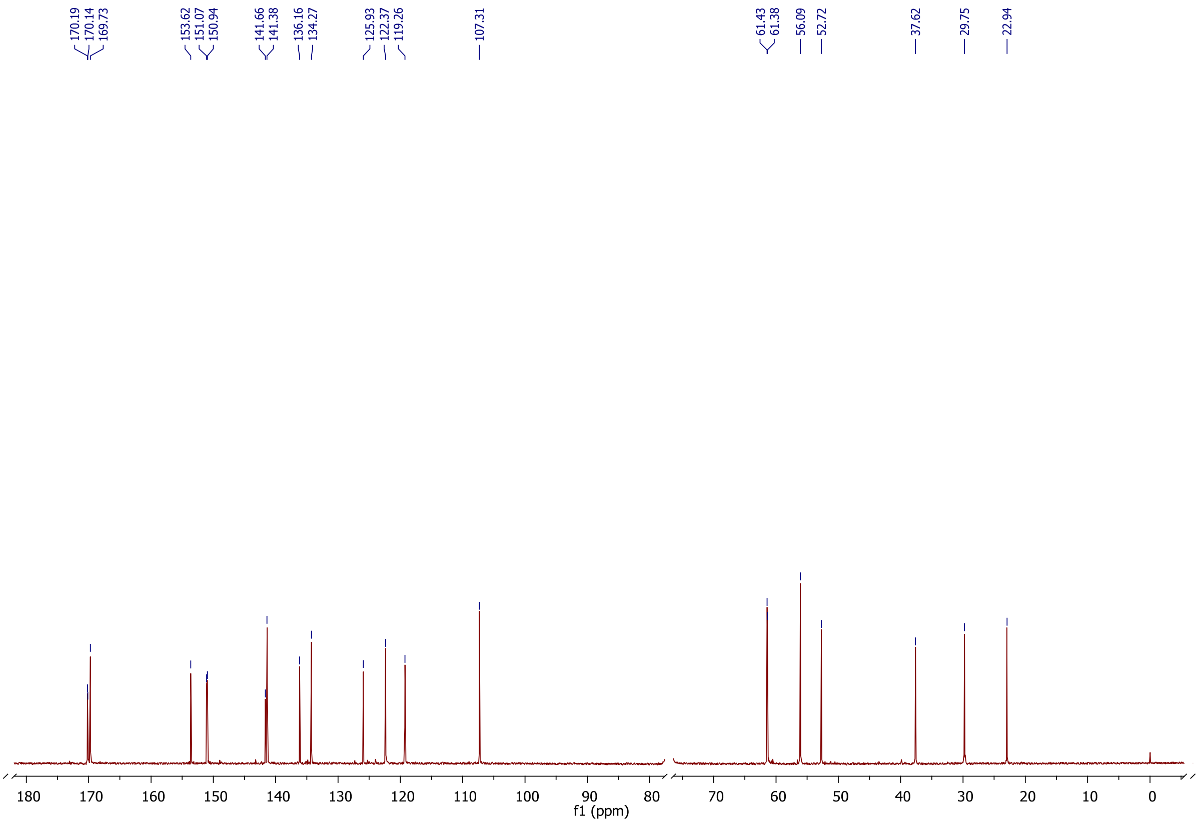

Figure S10. ^{1}H NMR of **2-NaCl**

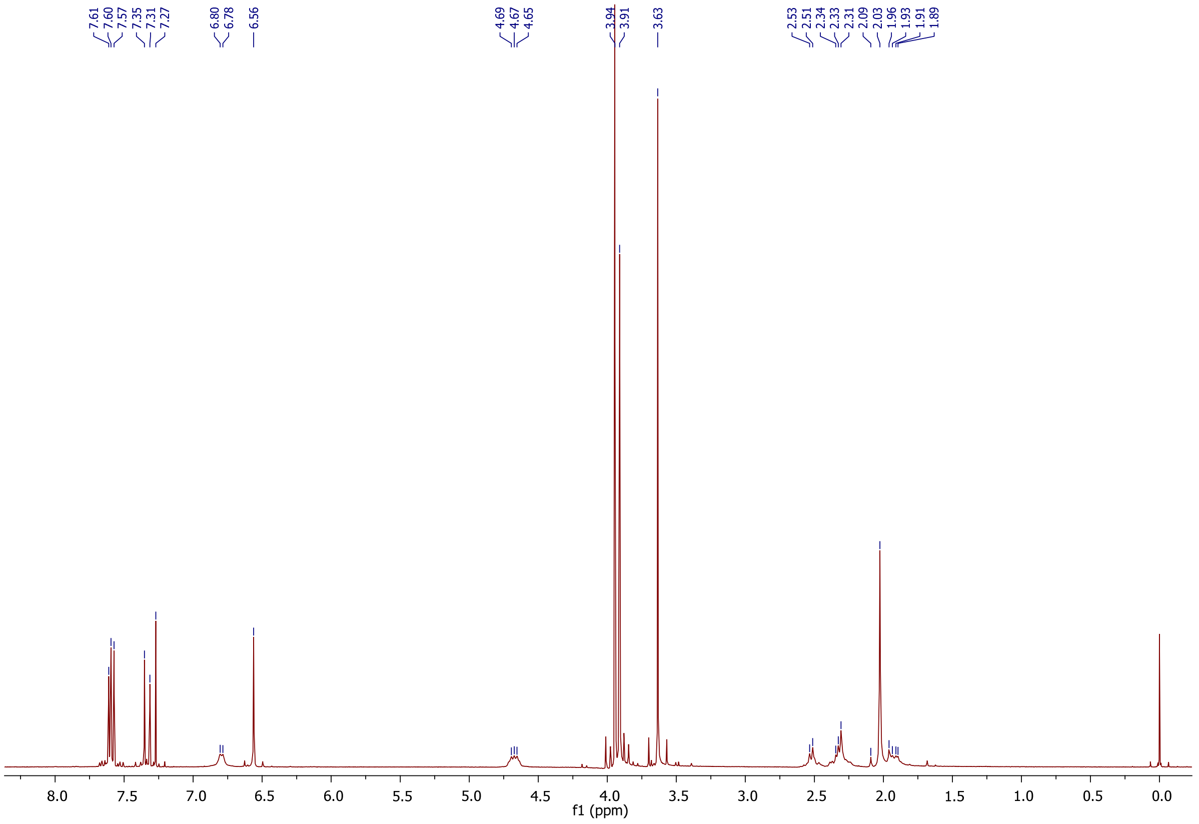

1.6. Figure S11. ^{13}C NMR of **2-KCl**

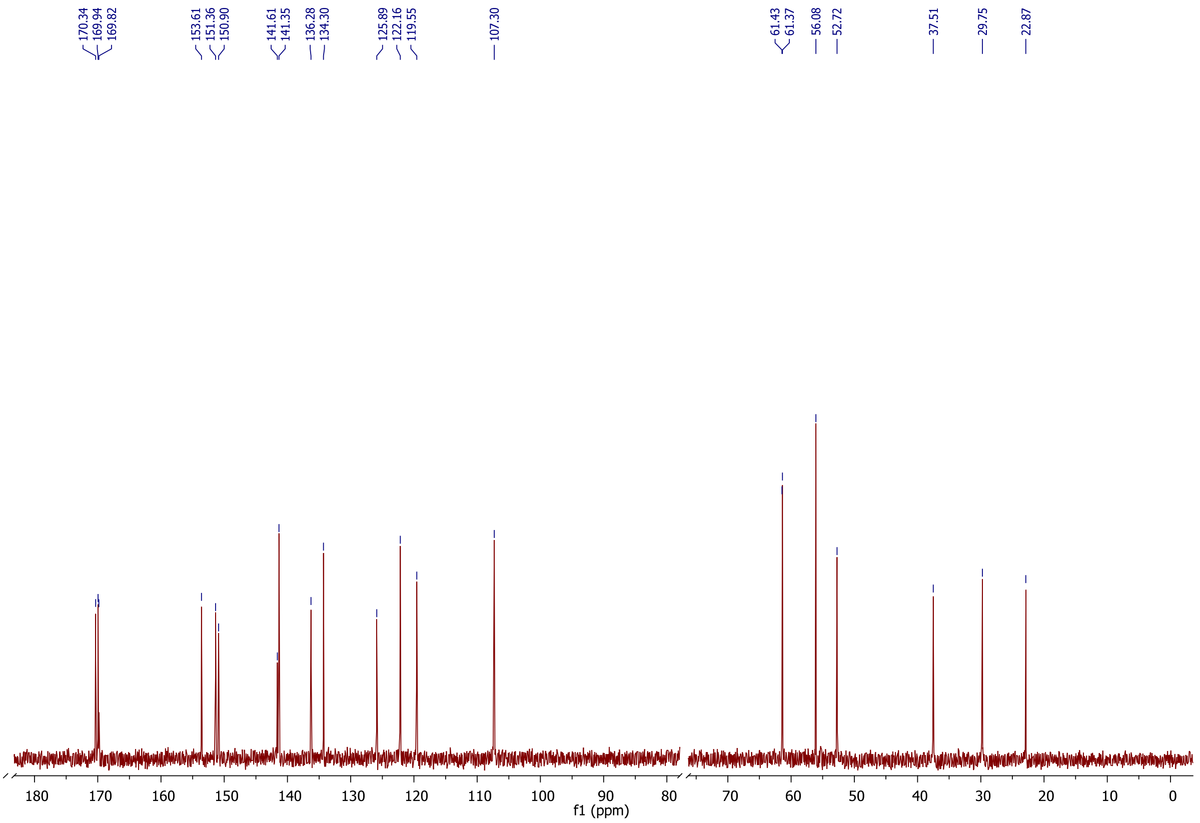

Figure S12. ^{1}H NMR of **2-KCl**

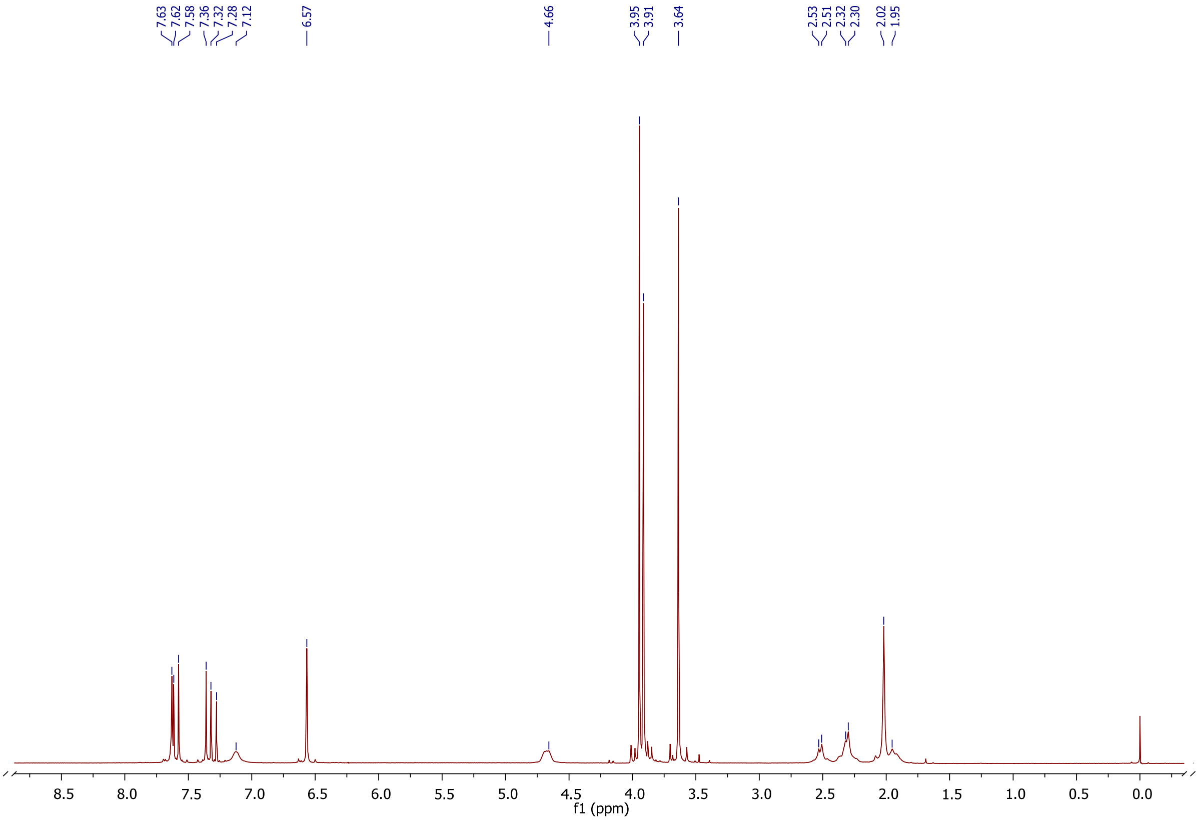

1.7. **Figure S13.** ^{13}C NMR of **3-LiCl**

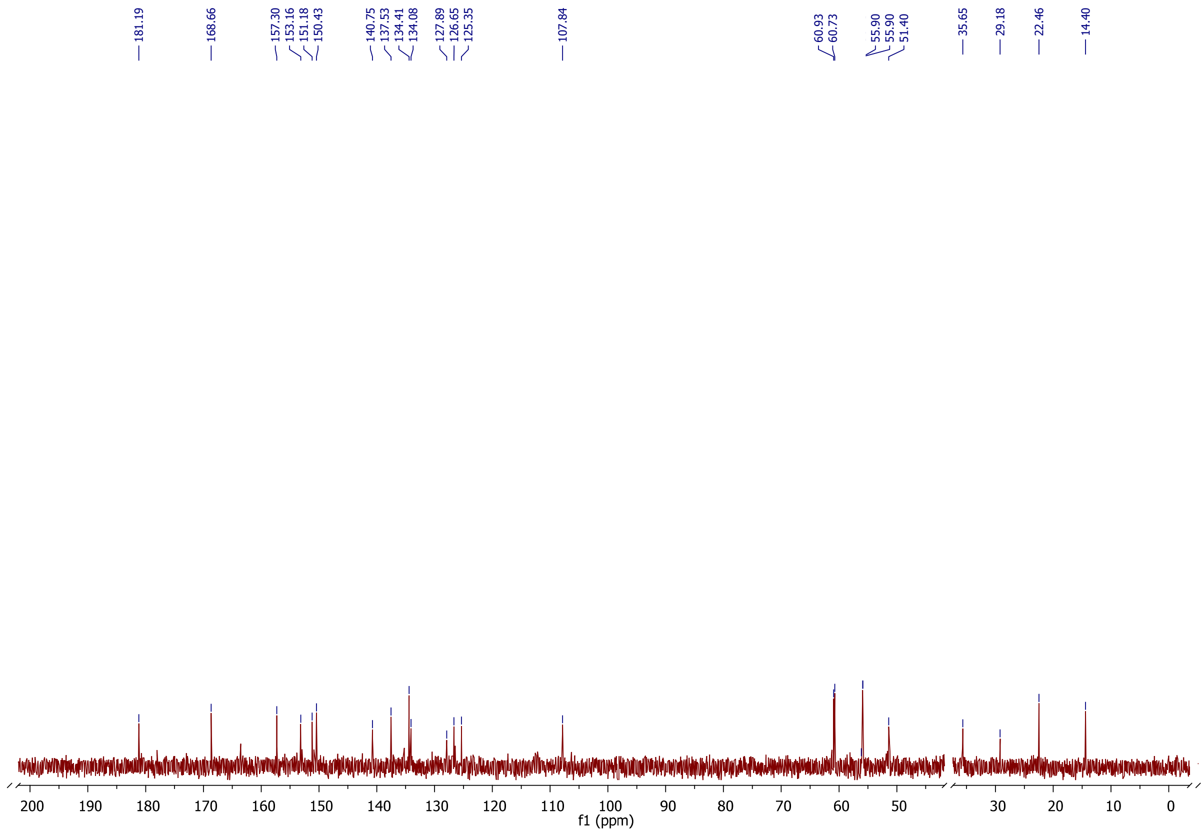

Figure S14. ^{1}H NMR of **3-LiCl**

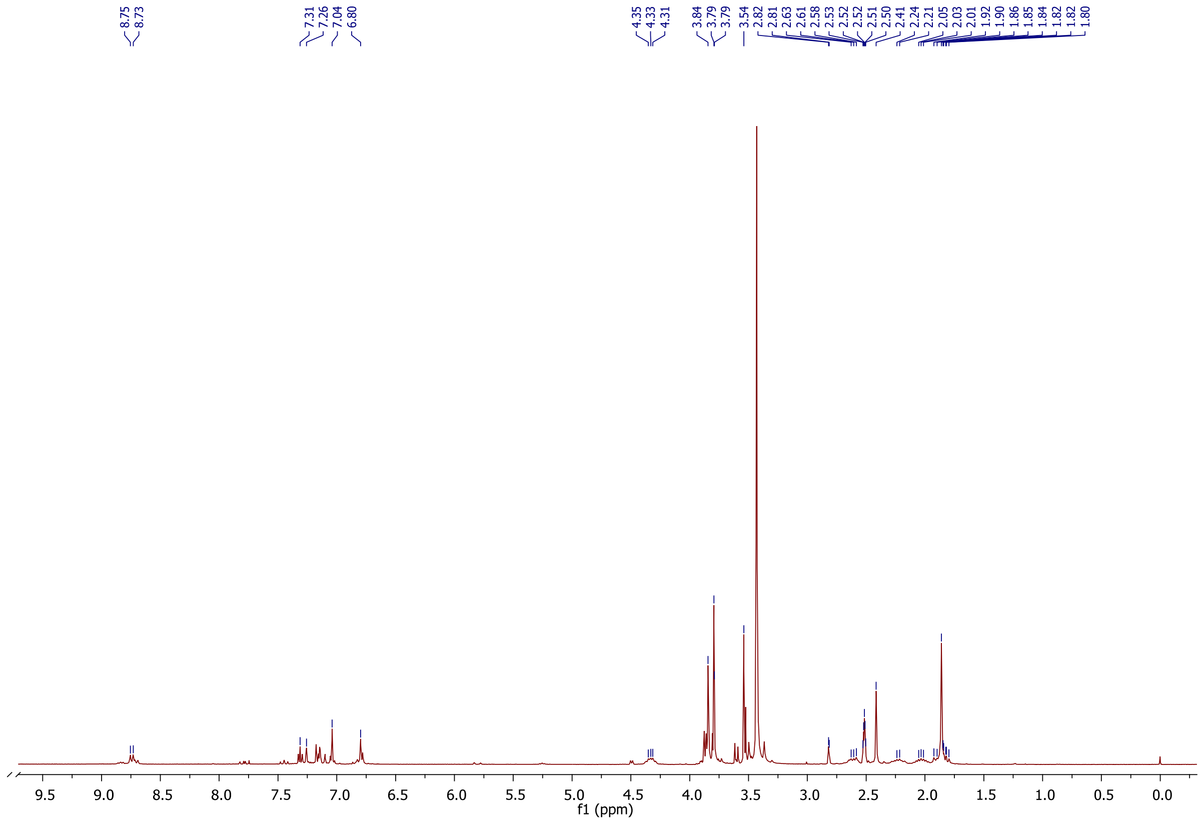

1.8. **Figure S15.** ^{13}C NMR of **3-NaCl**

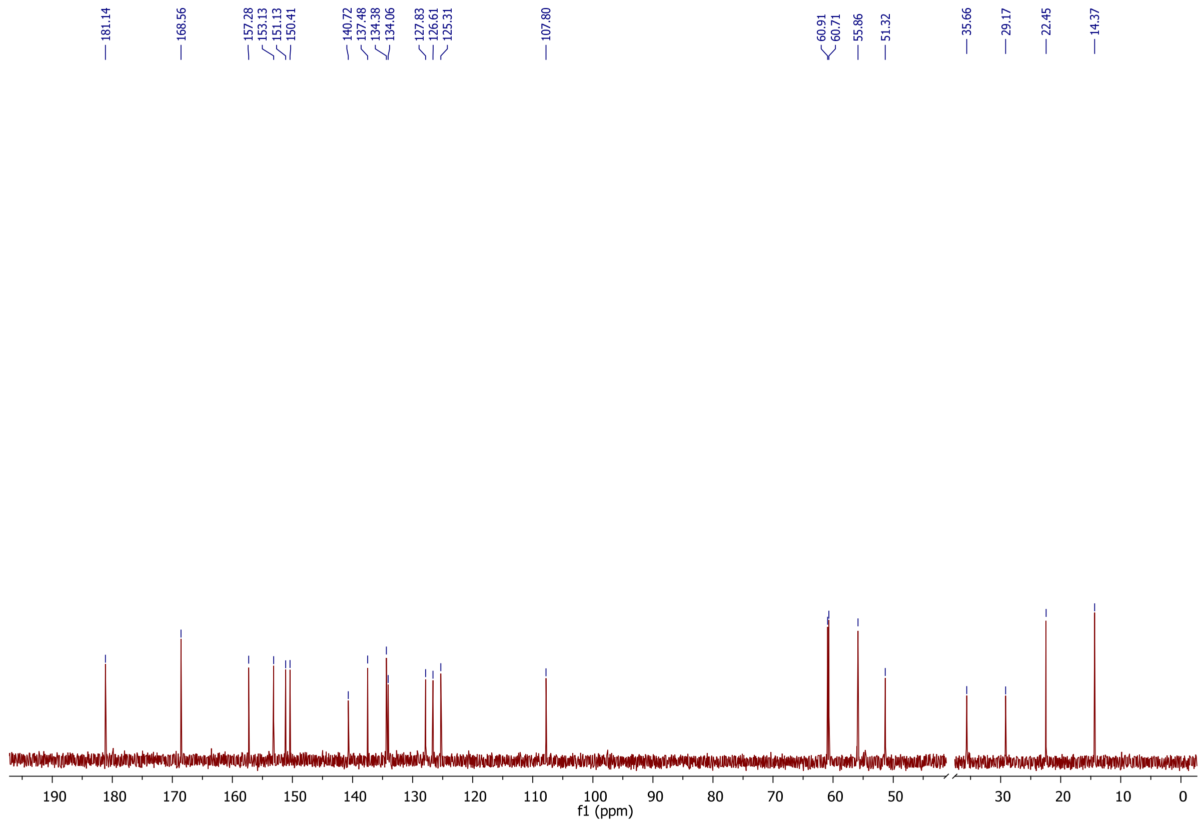

Figure S16. ^{1}H NMR of **3-NaCl**

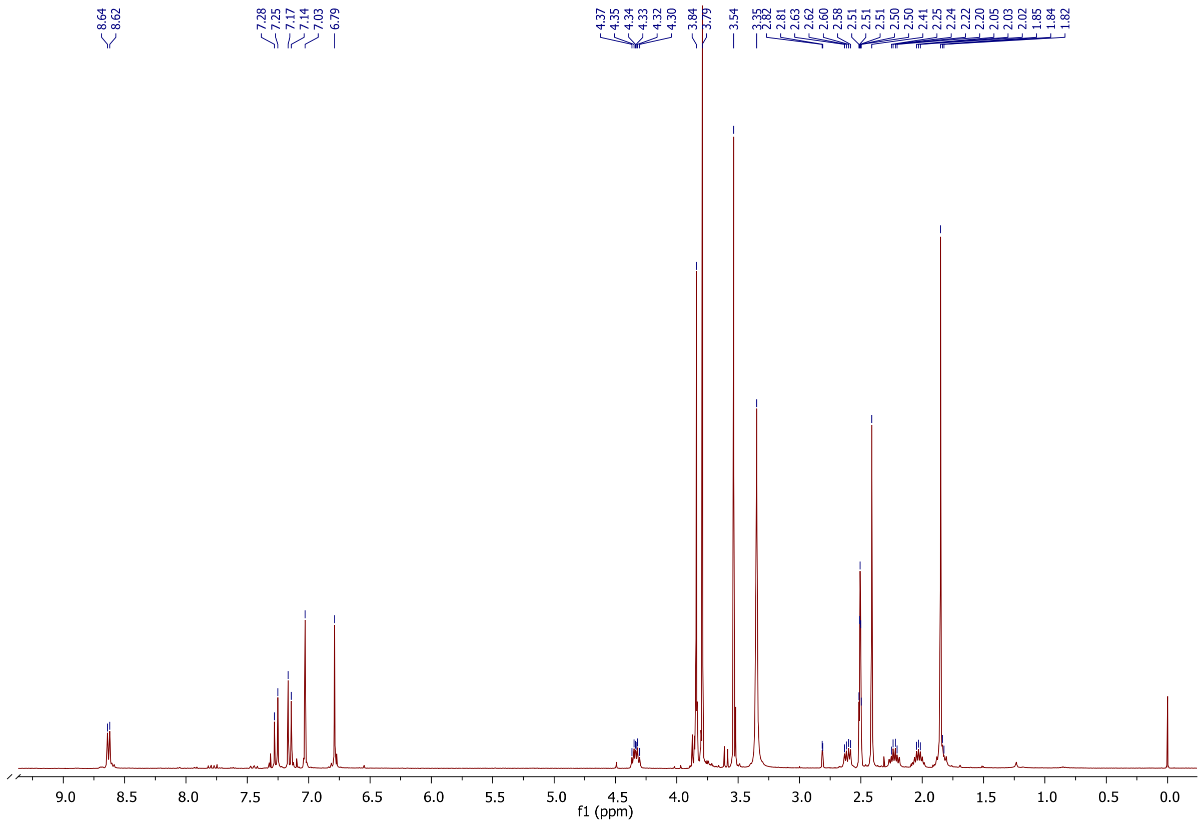

1.9. **Figure S17.** ^{13}C NMR of **3-KCl**

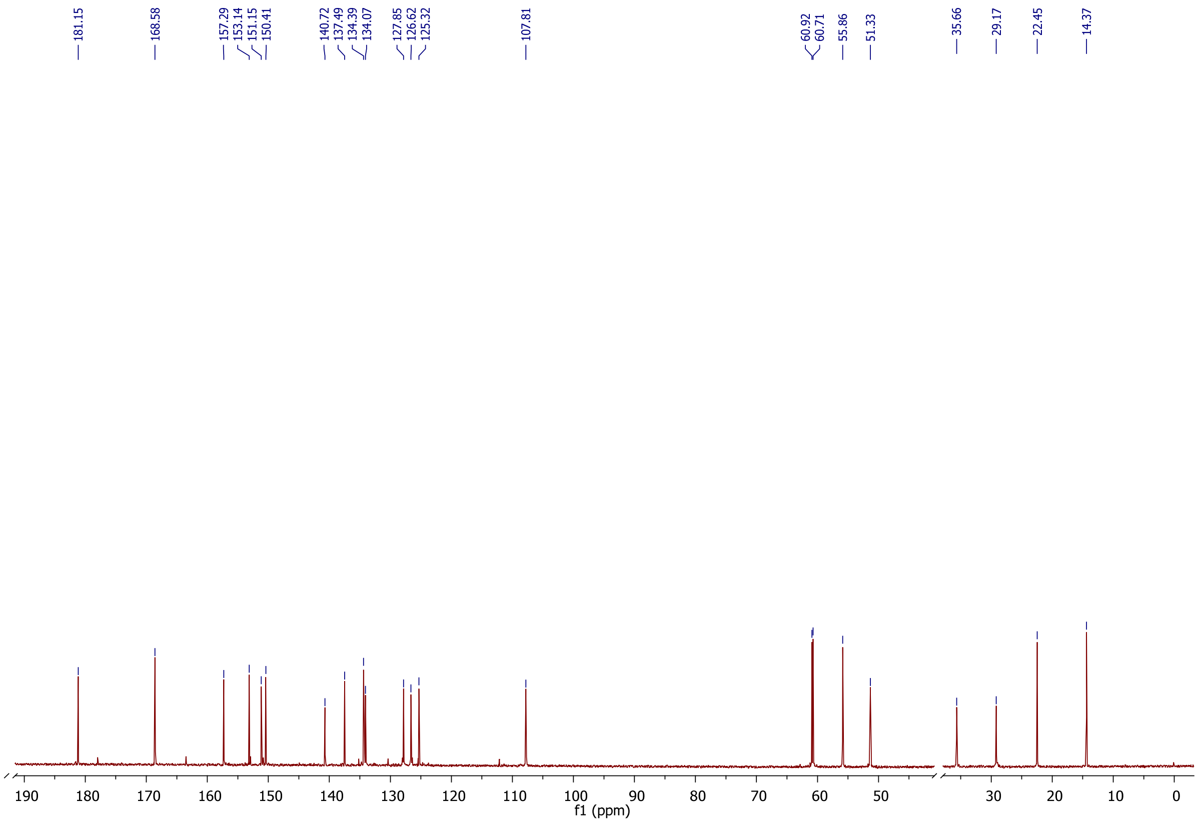

Figure S18. ^{1}H NMR of **3-KCl**

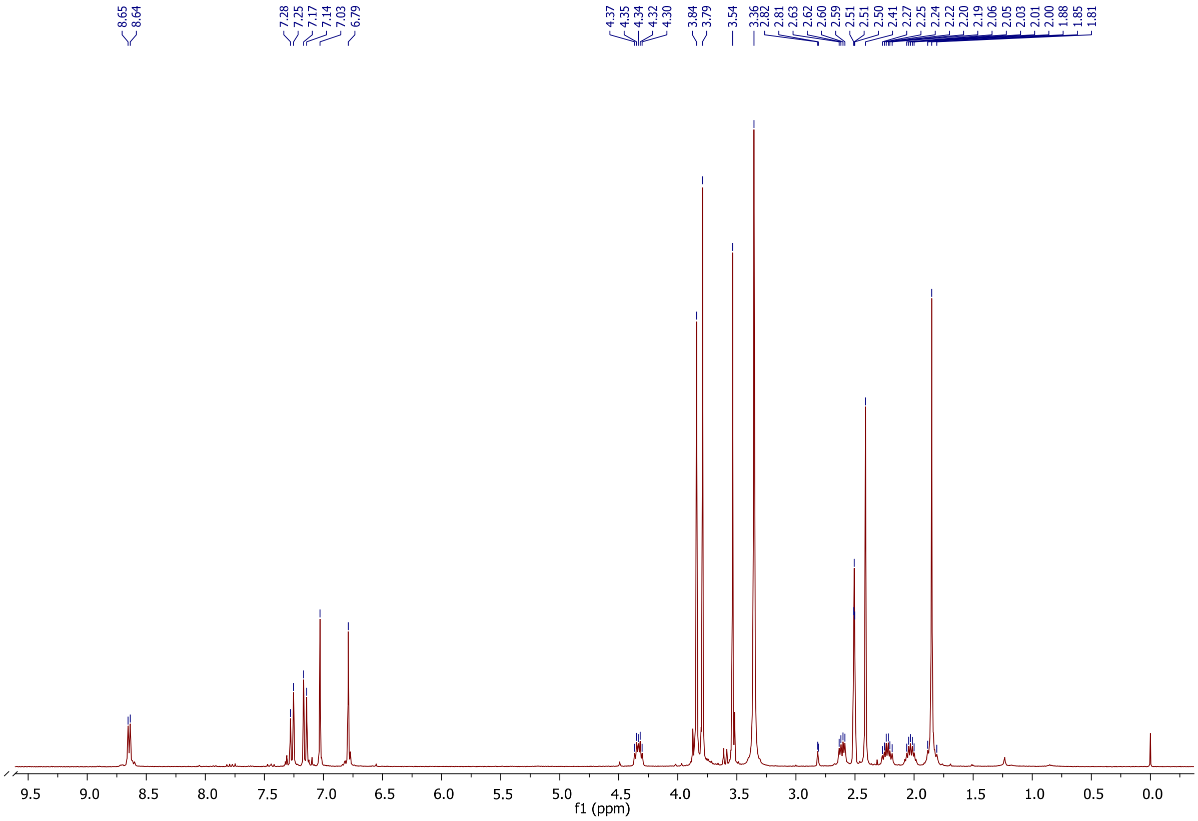

2. FT IR spectra of complexes

Figure S19. The FT IR spectra of **1**, **1-LiCl, 1-NaCl** and **1-KCl** in the range of 1750-1500cm^{-1}

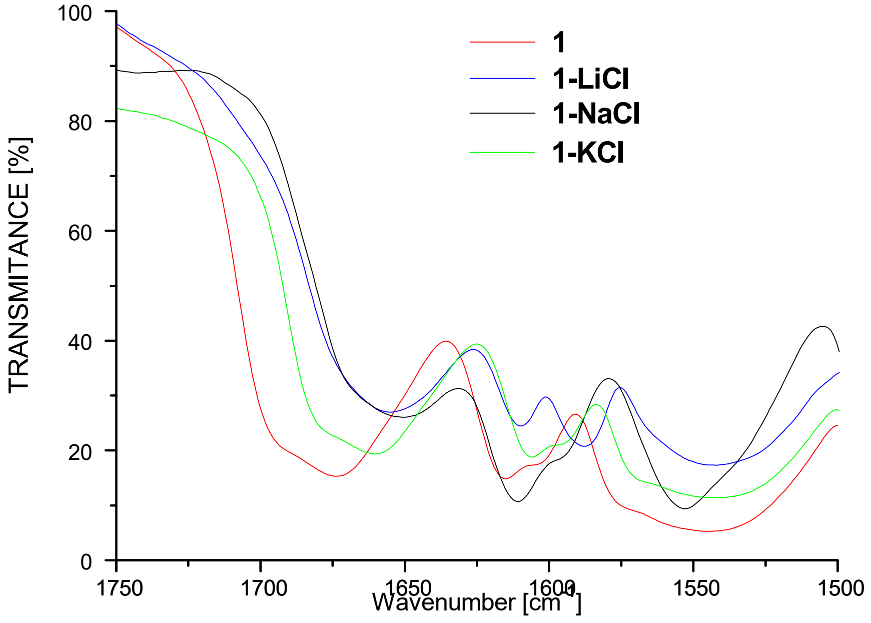

Figure S20. The FT IR spectra of **2**, **2-LiCl, 2-NaCl** and **2-KCl** in the range of 1750-1500cm^{-1}

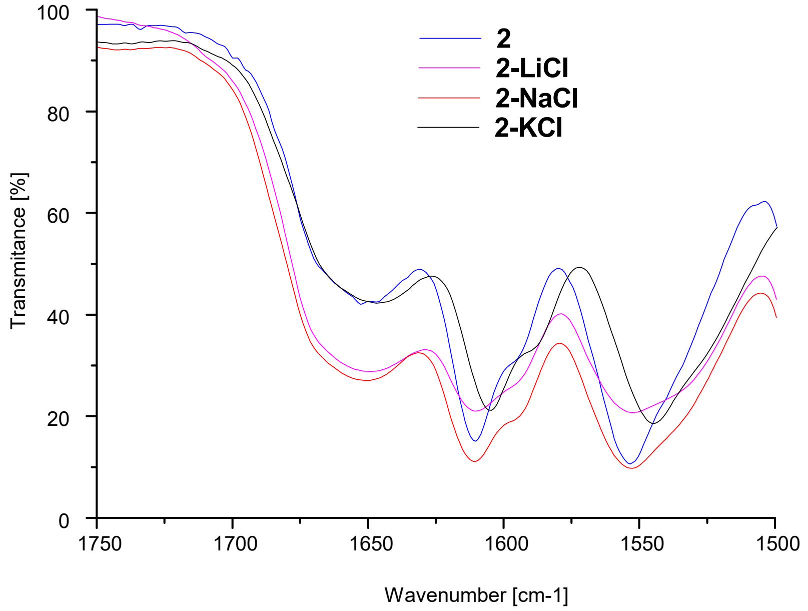

Figure S21. The FT IR spectra of **3**, **3-LiCl, 3-NaCl** and **3-KCl** in the range of 1750-1500cm^{-1}

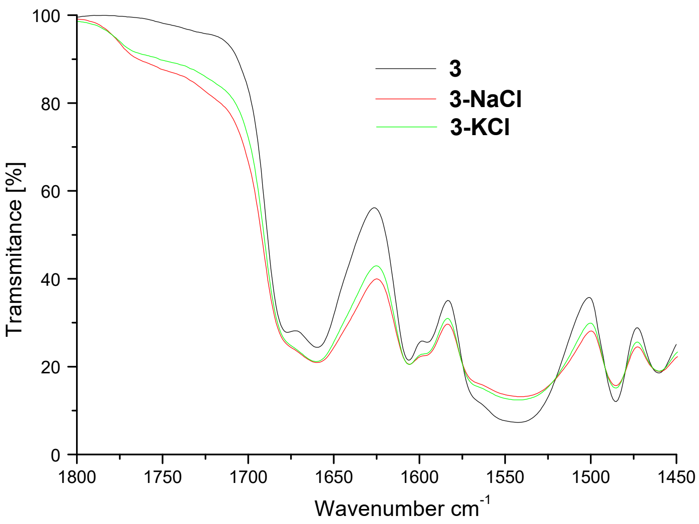

3. ESI MS mass spectra

Figure S22. The main peaks in the ESI mass spectra of the complexes of colchicine complexes **1-Li, 1-Na** and **1-K** (**1** = **M**) with monovalent cations chlorides measured at cv=30V.

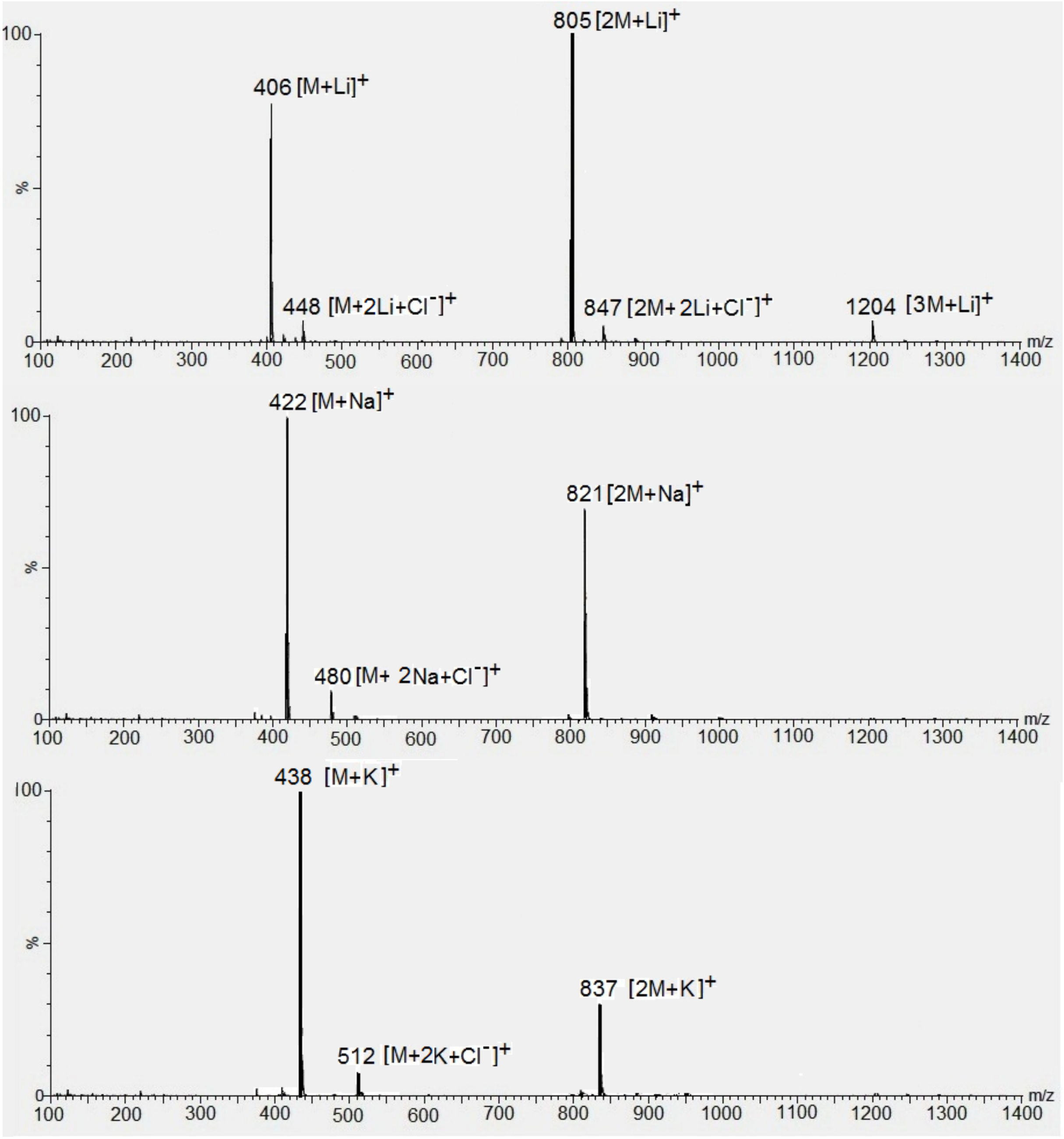

Figure S23. The main peaks in the ESI mass spectra of the complexes of colchiceine complexes **2-Li, 2-Na** and **2-K** with monovalent cations chlorides measured at cv=30V.

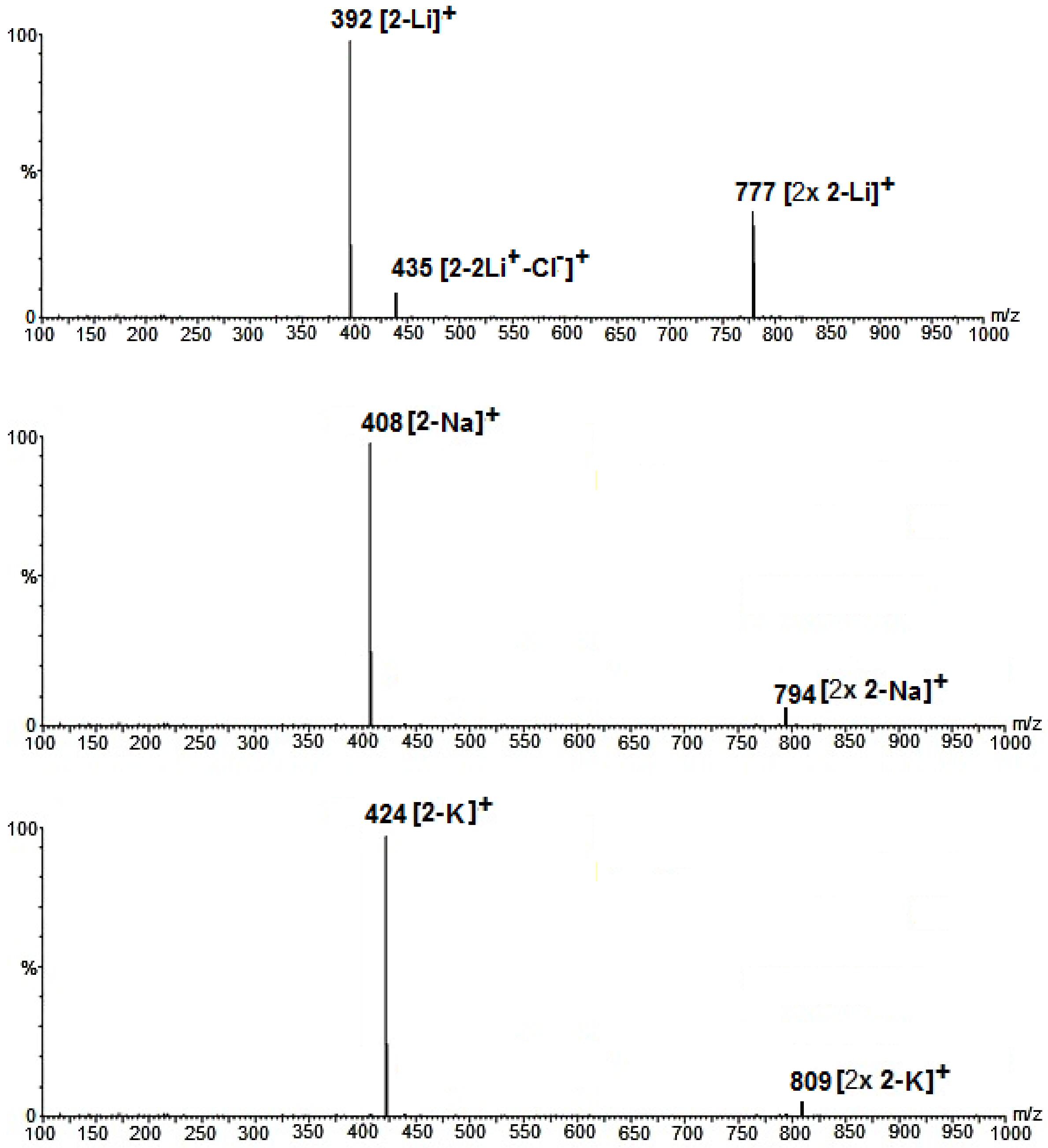

Figure S24. The main peaks in the ESI mass spectra of the complexes of 10-methylthiocolchicine complexes **3-Li, 3-Na** and **3-K** with monovalent cations chlorides measured at cv=30V.

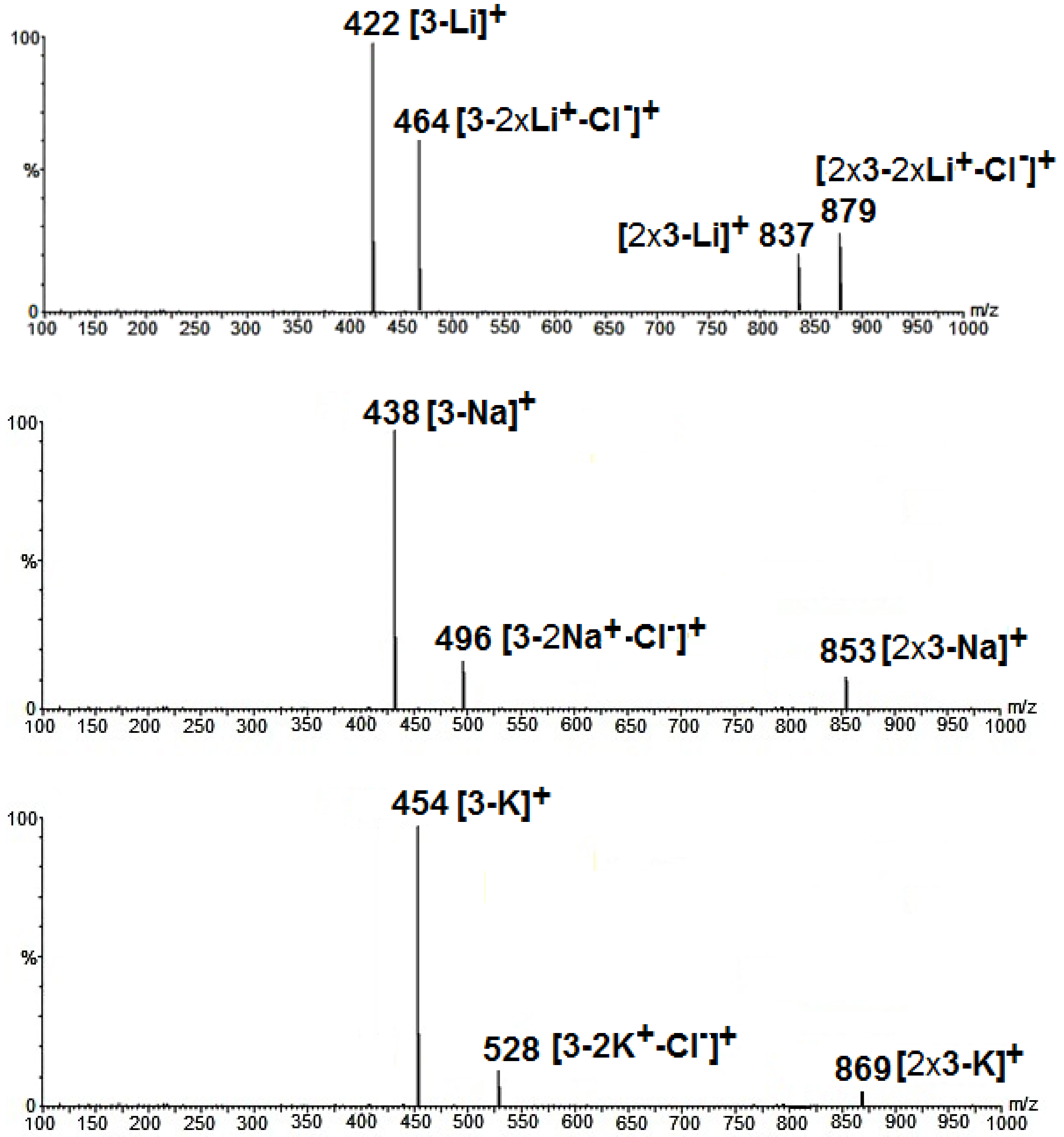

4. Fungicidal activity of complexes

4.1.

Table S1. The results of bioassay tests of colchicine **1**, colchiceine **2**, 10-methylthiolchicine **3** their complexes and respective salts against microfungi.

Compound	Fungial species							
	A. niger	*A. versicolor*	*P. variotii*	*P. funiculosum*	*T. viride*	*P. cyclopium*	*A. pullulans*	*Ch. globosum*
**1**	-	-	-	-	-	-	±	-
**1-LiCl**	-	±	-	-	±	±	-	-
**1-NaCl**	-	-	-	-	-	-	-	-
**1-KCl**	-	-	-	-	-	-	-	-
**2**	+	+	+	+	+	+	+	+
**2-LiCl**	-	-	-	+	-	-	+	+
**2-NaCl**	-	-	-	+	-	-	+	+
**2-KCl**	-	±	±	+	-	-	+	+
**3**	-	-	-	-	-	-	-	-
**3-LiCl**	-	-	-	-	-	+	+	-
**3-NaCl**	-	-	-	-	-	+	+	-
**3-KCl**	-	-	-	-	-	+	+	-
**LiCl**	-	-	-	-	-	-	-	-
**NaCl**	-	-	-	-	-	-	-	-
**KCl**	-	-	-	-	-	-	-	-

'-' – visible growth

' ± ' -

' + ' – no visible growth

4.2.

Table S2. The results of bioassay tests for minimal fungicidal concentration (MFC**[μg/mL]** and **[mMol/mL]**) against eight microfungi species

**Complex**	**MFC ([μg/mL] and [mMol/mL]) of different fungal species**							
	A. niger	*A. versicolor*	*P. variotti*	*P. funiculosum*	*T. viride*	*P. cyclopium*	*A. pullulans*	*Ch. globosum*
**1**	>4000	>4000	>4000	>4000	>4000	>4000	1.0±0.0 [$2.5\cdot10^{-12}$]	>4000
**1-LiCl**	>4000	>4000	>4000	>4000	>4000	>4000	>4000	>4000
**1-NaCl**	>4000	>4000	>4000	>4000	>4000	>4000	>4000	>4000
**1-KCl**	>4000	>4000	>4000	>4000	>4000	>4000	>4000	>4000
**2**	1000±0.0 [$2.6\cdot10^{-10}$]	1000±0.0 [$2.6\cdot10^{-10}$]	500±0.0 [$1.3\cdot10^{-10}$]	2000±0.0 [$5.2\cdot10^{-10}$]	1000±0.0 [$2.6\cdot10^{-10}$]	1000±0.0 [$2.6\cdot10^{-10}$]	4.0±0.0 [$1.\cdot10^{-12}$]	4000±0.0 [$1.\cdot10^{-10}$]
**2-LiCl**	4000±0.0 [$9.3\cdot10^{-10}$]	4000±0.0 [$9.3\cdot10^{-10}$]	4000±0.0 [$9.3\cdot10^{-10}$]	2000±0.0 [$4.6\cdot10^{-10}$]	4000±0.0 [$9.3\cdot10^{-10}$]	4000±0.0 [$9.3\cdot10^{-10}$]	260±0.0 [$6.0\cdot10^{-11}$]	130±0.0 [$3.0\cdot10^{-11}$]
**2-NaCl**	4000±0.0 [$9\cdot10^{-10}$]	4000±0.0 [$9\cdot10^{-10}$]	4000±0.0 [$9\cdot10^{-10}$]	2000±0.0 [$4.5\cdot10^{-10}$]	4000±0.0 [$9\cdot10^{-10}$]	4000±0.0 [$9\cdot10^{-10}$]	500±0.0 [$1.1\cdot10^{-10}$]	130±0.0 [$2.9\cdot10^{-11}$]
**2-KCl**	4000±0.0 [$8.7\cdot10^{-10}$]	4000±0.0 [$8.7\cdot10^{-10}$]	4000±0.0 [$8.7\cdot10^{-10}$]	2000±0.0 [$4.3\cdot10^{-10}$]	4000±0.0 [$8.7\cdot10^{-10}$]	4000±0.0 [$8.7\cdot10^{-10}$]	1000±0.0 [$2.2\cdot10^{-10}$]	130±0.0 [$2.8\cdot10^{-11}$]
**3**	>4000	>4000	>4000	>4000	>4000	>4000	>4000	>4000
**3-LiCl**	4000±0.0 [$8.7\cdot10^{-10}$]	4000±0.0 [$8.7\cdot10^{-10}$]	4000±0.0 [$8.7\cdot10^{-10}$	4000±0.0 [$8.7\cdot10^{-10}$]	4000±0.0 [$8.7\cdot10^{-10}$]	4000±0.0 [$8.7\cdot10^{-10}$]	4000±0.0 [$8.7\cdot10^{-10}$]	4000±0.0 [$8.7\cdot10^{-10}$]
**3-NaCl**	4000±0.0 [$8.4\cdot10^{-10}$]	4000±0.0 [$8.4\cdot10^{-10}$]	4000±0.0 [$8.4\cdot10^{-10}$]	4000±0.0 [$8.4\cdot10^{-10}$]	4000±0.0 [$8.4\cdot10^{-10}$]	4000±0.0 [$8.4\cdot10^{-10}$]	4000±0.0 [$8.4\cdot10^{-10}$]	4000±0.0 [$8.4\cdot10^{-10}$]
**3-KCl**	4000±0.0 [$8.2\cdot10^{-10}$]	4000±0.0 [$8.2\cdot10^{-10}$]	4000±0.0 [$8.2\cdot10^{-10}$]	4000±0.0 [$8.2\cdot10^{-10}$]	4000±0.0 [$8.2\cdot10^{-10}$]	4000±0.0 [$8.2\cdot10^{-10}$]	4000±0.0 [$8.2\cdot10^{-10}$]	4000±0.0 [$8.2\cdot10^{-10}$]
**chalcone**	130±0.0 [$6.2\cdot10^{-11}$]	4000±0.0 [$1.9\cdot10^{-12}$]	2000±0.0 [$9.6\cdot10^{-10}$]	260±0.0 [$1.2\cdot10^{-10}$]	1000±0.0 [$4.8\cdot10^{-10}$]	500±0.0 [$2.4\cdot10^{-10}$]	1000±0.0 [$4.8\cdot10^{-10}$]	260±0.0 [$1.2\cdot10^{-10}$]
**IPBC** (fungicide)*	5.2±0.0 [$1.8\cdot10^{-12}$]	5.2±0.0 [$1.8\cdot10^{-12}$]	5.2±0.0 [$1.8\cdot10^{-12}$]	5.2±0.0 [$1.8\cdot10^{-12}$]	0.2±0.0 [$7\cdot10^{-12}$]	5.2±0.0 [$1.8\cdot10^{-12}$]	5.2±0.0 [$0.9\cdot10^{-12}$]	10±0.0 [$3.6\cdot10^{-10}$]

Physicochemical properties predictions

References to LogP

[1] ESOL J.S. Delaney: Estimating Aqueous Solubility Directly from Molecular Structure. *J. Chem. Inf. Comput. Sci.* **44**,3, 1000–1005.(2004)
Ali [2] J. Ali, P. Camilleri, M. B. Brown, A.J. Hutt, S. B. Kirton *In Silico* Prediction of Aqueous Solubility Using Simple QSPR Models: The Importance of Phenol and Phenol-like Moieties. *J. Chem. Inf. Model.* **52**, 11, 2950–2957 (2012)
SILICOS-IT[3] fragmental metod calculated by FILTER-IT program, version 1.0.2., courtesy of SILICOS-IT, http://www.silicos-it.com

[2] Log p (iLOGP) Daina, A.; Michielin, O.; Zoete, V.; iLOGP: a simple, robust, and efficient description of n-octanol/water parti-tion coefficient for drug design using the GB/SA approach. J Chem Inf Model. 2014 22:54:12:3284–301. doi: 10.1021/ci500467k].

[3] Log P (XLOGP3) Atomistic and knowledge-based method calculated by XLOGP program, version 3.2.2., courtesy of CCBG, Shanghai Institute of Organic Chemistry.

[4] Log P (WLOGP) Atomistic method implemented from: Wildman, S.A.; Crippen, G.M.; Prediction of Physicochemical Pa-rameters by Atomic Contributions. J. Chem. Inf. Model 1999 39:5. Topological method implemented from: Morigichi.I.; Hirono, S.; Liu Q.; Nakagome, I.; Matsushita Y.; Simple method of calculating octanol/water partition coefficient. Chem. Pharm. Bull 1992 40:1:127-130. Lipinski P.A. Lombardo F.; Dominy B. W.; Feeney P. J.; Experimental and computational ap-proaches to estimate solubility and permeability in drug discovery and development settings. Adv. Drug. Deliv. Rev. 2001 1;46(1-3):3-26. doi: 10.1016/s0169-409x(00)00129-0.

[5] Log P (MLOGP) Hybrid fragment al/topological method calculated by FILTER-IT program, version 1.0.2, courtesy of SILI-COS-IT, hptt://www.silicos-it.com., http://www.swissadme.ch/ [accessed 20.11.2025]

[6] Log P (SILICOS-IT) http://www.swissadme.ch/ [accessed 20.11.2025]

[7] Consensus Log P average of all five predictions 2–6. http://www.swissadme.ch/ [accessed 20.11.2025]

[8] LogP Protox II. Banerjee P., Eckert A. O., Schrey A.K., Preissner R., ProTox-II: a webserver for the prediction of toxicity of chemicals. Nucleic Acids Res. 2018 Jul 2; 46(Web Server issue): W257–W263. doi: 10.1093/nar/gky318 http://www.swissadme.ch/ [accessed 20.11.2025]

Table S3. Physicochemical and ADME data of synthesized compounds and standard reference compounds as predicted by SwissADME web tool.

Cpd.	MW	RB	Lipinski's violation	GI	Leadlike	Druglikeness
**1**	399.5	6	0	high	No	Yes
**1-LiCl**	441.8	5	0	high	No	Yes
**1-NaCl**	457.9	5	0	high	No	Yes
**1-KCl**	473.9	5	0	high	No	Yes
**2**	385.4	5	0	high	no	yes
**2-LiCl**	426.8	5	0	high	no	yes
**2-NaCl**	442.8	5	0	high	no	yes
**2-KCl**	459.9	5	0	high	no	yes
**3**	415.5	6	0	high	no	yes
**3-LiCl**	457.9	5	0	high	No	Yes
**3-NaCl**	473.9	5	0	high	No	Yes
**3-KCl**	490.0	5	0	high	No	Yes
**IPBC**	281.1	6	0	high	yes	yes
**chalcone**	208.3	3	0	high	no	yes
**doxoribicin**	543.5	5	3	low	No	No

4.1.

Table S4. Oral toxicity prediction results obtained by Protox II tool [8].

Comp.	Molecuar weight	Number of hydrogen bond acceptors	Number of hydrogen bond donors	Number of atoms	Number of bonds	Molecular refractivuty	Topological Polar Surface Area	Predicted LD_{50} mg/kg	Predicted toxicity Active 1-6 inactive	Predicted cytotoxicity
**1**	399	**6**	**1**	29	31	109.36	**83.09**	6	2	**active**
**1-LiCl**	442	**6**	**1**	30	32	109.35	**83.09**	19	2	**active**
**1-NaCl**	458	**6**	**1**	30	32	109.35	**83.09**	19	2	**active**
**1-KCl**	474	**6**	**1**	30	32	109.35	**83.09**	19	2	**active**
**2**	385	**6**	**2**	30	32	104.89	**94.09**	6	2	**active**
**2-LiCl**	428	**6**	**1**	30	32	103.84	**94.06**	400	4	**active**
**2-NaCl**	444	**6**	**1**	30	32	102.92	**83.09**	500	4	**active**
**2-KCl**	460	**6**	**1**	30	32	102.92	**83.09**	1000	4	**active**
**3**	415	**5**	**1**	30	32	114.59	**99.16**	19	2	**active**
**3-LiCl**	458	**5**	**1**	30	32	114.58	**99.16**	19	2	**active**
**3-NaCl**	474	**5**	**1**	30	32	120.21	**99.16**	19	2	**active**
**3-KCl**	490	**5**	**1**	30	32	120.21	**99.16**	19	2	**active**
chalcone	208	**1**	**0**	16	17	66.25	**17.07**	1048	4	**inactive**
IPBC	281	**2**	**1**	11	10	51.36	**38.33**	1000	4	**inactive**
doxorubicin	544	**12**	**6**	39	40	132.66	**206.07**	205	3	**active**

5. Lipophilicity of complexes

Fig. S25. Molecular lipophilicity potentials of alkaloids: **1**, **2**, **3** and complexes with lithium chloride

**Alkaloid**	**Complex**
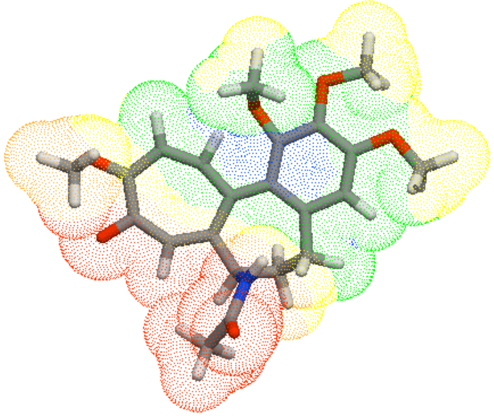<br>**1**	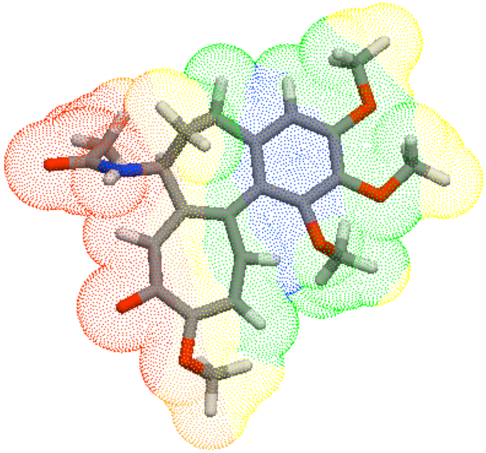<br>**1-LiCl**
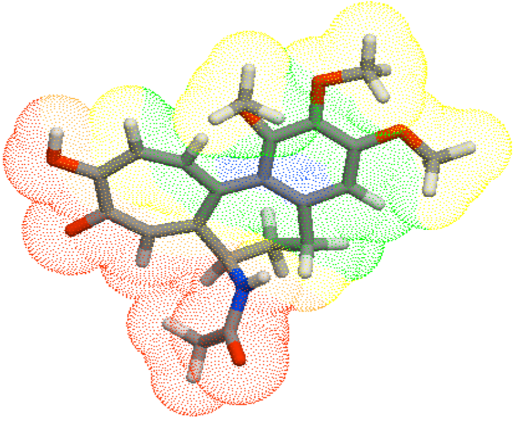<br>**2**	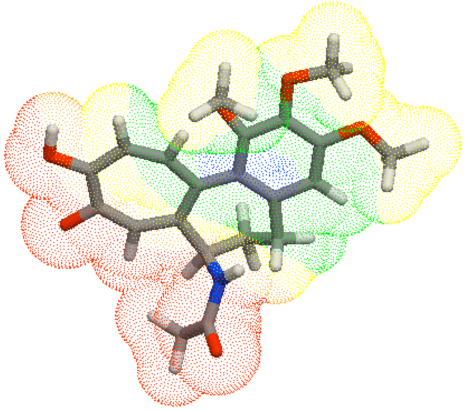<br>**2-LiCl**
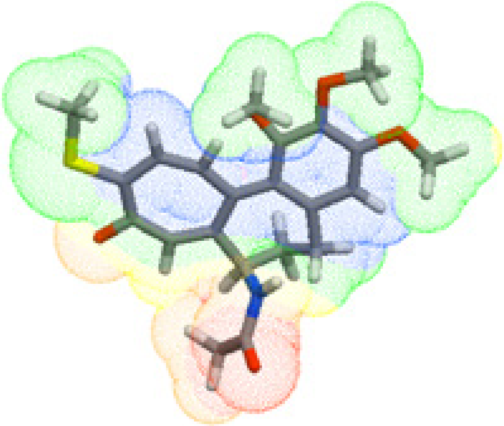<br>**3**	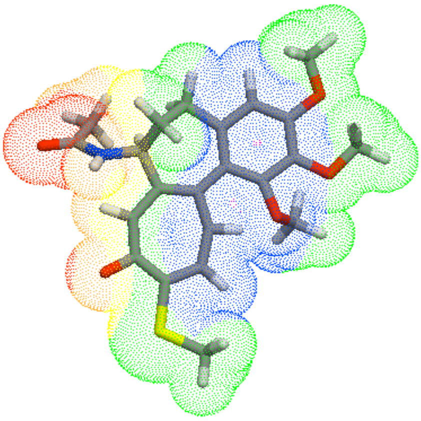<br>**3-LiCl**

7. DFT calculations

Table S5. Extended version of Table 1 with calculated energies (counterpoise energy, BSSE, sum of monomers energy, counterpoise uncorrected, corrected interaction energies in vacuum and energy, sum of monomers energy and interaction energy in methanol) for the studied interaction schemes of 10-methyltiocolchicine with Li cation.

	Vacuum					Methanol		
Complex	Counterpoise corrected energy [Hartree]	BSSE energy [Hartree]	Sum of monomers [hartree]	Interaction Energy (raw) [kcal/mol]	Interaction Energy (corrected) [kcal/mol]	Energy [Hartree]	Sum of monomers [hartree]	Interaction Energy [kcal/mol]
1:1 Type **A**	-1689.002347	0.001259	-1688.860792	-89.6	-88.8	-1689.094212	-1689.080727	-8.5
1:1 Type **B**	-1688.970684	0.000990	-1688.872537	-62.2	-61.6	-1689.093107	-1689.089053	-2.5
1:1 Type **C**	-1689.007574	0.000558	-1688.853673	-96.9	-96.6	-1689.092578	-1689.076927	-9.8
2:1 Type **D**	-3370.686127	0.005189	-3370.475934	-135.2	-131.9	-3370.767293	-3370.726770	-25.4
2:1 Type **E**	-3370.669295	0.005295	-3370.446453	-143.2	-139.8	-3370.738978	-3370.698002	-25.7

Table S6. Extended version of Table 1 (obtained at the wB97XD/Def2TZVPP level of theory) for 1:1 stoichiometry complexes with calculated energies (counterpoise energy, BSSE, sum of monomers energy, counterpoise uncorrected, corrected interaction energies in vacuum and energy, sum of monomers energy and interaction energy in methanol) for the studied interaction schemes of 10-methyltiocolchicine with Li cation.

Vacuum					Methanol		
Counterpoise corrected energy [Hartree]	BSSE energy [Hartree]	Sum of monomers [hartree]	Interaction Energy (raw) [kcal/mol]	Interaction Energy (corrected) [kcal/mol]	Energy [Hartree]	Sum of monomers [hartree]	Interaction Energy [kcal/mol]
-1689.897477	0.000600	-1689.752727	-91.2	-90.8	-1689.988104	-1689.971091	-10.7
-1689.864151	0.000553	-1689.764682	-62.8	-62.4	-1689.985424	-1689.979482	-3.7
-1689.897245	0.000607	-1689.742008	-97.8	-97.4	-1689.981839	-1689.963724	-11.4

Table S7. Selected geometric parameters and calculated Mulliken point charges for structures A-E of **3-Li** complexes. (CA : coordinating atom)

Complex	Cation Mulliken partial charge	Coordinating atom (CA)	Coordinating atom Mulliken partial charge	Distance between coordinating atom and cation [Å]
1:1 Type A	0.583	O1	-0.283	1.988
		O2	-0.287	2.087
		O4	-0.422	1.904
1:1 Type B	0.714	O1	-0.336	1.897
		O2	-0.387	1.896
1:1 Type C	0.630	O4	-0.520	1.874
		O5	-0.471	1.850
2:1 Type D	0.018	O1a	-0.092	2.156
		O2a	-0.304	2.172
		O4a	-0.298	1.899
		O4b	-0.397	1.850
2:1 Type E	-0.092	O4a	-0.488	1.987
		O5a	-0.265	2.088
		O4b	-0.410	2.087
		O5b	-0.426	2.014

Table S8. Atomic coordinates of optimized 1:1 stoichiometry colchicine complexes with lithium cation.

1:1:1 stoichiometry colchicine complexes atomic coordinates									
	Type **A**			Type **B**			Type **C**		
Atom	x	y	z	x	y	z	x	y	z
C	5.5233	-0.5139	-1.6843	6.0095	1.2606	-1.1974	6.1168	1.6120	-1.0046
O	4.7493	0.3819	-0.9014	5.3448	0.1232	-0.6588	5.5001	0.4132	-0.5698
C	3.4091	0.2833	-0.9589	4.0088	0.1732	-0.5175	4.1655	0.3999	-0.4017
C	2.6982	-0.6670	-1.6953	3.2044	1.2729	-0.8105	3.3385	1.5045	-0.5919
C	1.3118	-0.7404	-1.5817	1.8168	1.2227	-0.6803	1.9553	1.3883	-0.4901
C	0.5810	0.1943	-0.8304	1.1692	0.0442	-0.2745	1.3521	0.1596	-0.1908
C	1.2923	1.1867	-0.1428	1.9963	-1.0277	0.0991	2.2094	-0.9417	0.0954
O	0.8076	2.1177	0.7467	1.4921	-2.2418	0.5729	1.6322	-2.1146	0.4546
C	-0.5734	2.4281	0.9531	0.8418	-2.1620	1.8591	2.1720	-2.8614	1.5419
C	2.6940	1.1804	-0.1564	3.3864	-0.9738	-0.0076	3.6006	-0.8317	-0.0241
O	3.3419	1.9398	0.8005	4.1119	-2.1096	0.3365	4.3578	-1.9516	0.1011
C	3.8722	3.1877	0.3266	5.0220	-1.9333	1.4443	5.3574	-1.9571	1.1173
C	-0.8647	-0.0620	-0.5970	-0.3100	-0.1487	-0.2745	-0.1174	-0.0799	-0.1583
C	-1.7959	0.6942	-1.2546	-0.7398	-1.2240	-1.0187	-0.5243	-1.2460	-0.8116
C	-3.2093	0.7509	-1.1388	-1.9955	-1.8763	-1.1273	-1.7275	-1.9610	-0.8910

C	-4.0199	0.2375	-0.1585	-3.0436	-1.8577	-0.2442	-2.9408	-1.8015	-0.2445
S	-5.7455	0.4900	-0.0609	-4.4935	-2.8214	-0.3761	-4.3267	-2.8418	-0.4563
C	-6.1036	1.3083	-1.6356	-4.3539	-3.4860	-2.0545	-3.6933	-4.1378	-1.5507
C	-3.5779	-0.5781	1.0001	-3.0692	-1.0591	1.0039	-3.2296	-0.7203	0.6679
O	-4.3395	-0.7266	1.9501	-3.8044	-1.3941	1.9255	-4.4129	-0.5823	1.1104
C	-2.3388	-1.3493	0.9511	-2.3843	0.2297	1.0349	-2.2766	0.2713	1.0293
C	-1.1657	-1.1635	0.2854	-1.2162	0.6833	0.4951	-1.0551	0.7185	0.5661
C	-0.1820	-2.3265	0.3584	-1.0128	2.1887	0.7019	-0.9193	2.2216	0.8794
C	0.0846	-2.8999	-1.0488	0.3590	2.8168	0.4854	0.4389	2.8898	0.7617
C	0.5534	-1.9102	-2.1426	1.0092	2.4685	-0.8555	1.1297	2.6336	-0.5821
N	1.1263	-2.1705	1.0375	-1.9936	2.8536	-0.1611	-1.8181	2.9597	-0.0481
C	1.6515	-1.2954	1.9060	-2.5203	4.0699	0.1977	-3.1516	3.1436	-0.0074
C	3.0493	-1.6094	2.3728	-3.6001	4.6094	-0.6987	-3.7300	4.0340	-1.0673
O	1.0925	-0.2538	2.2956	-2.1285	4.6619	1.1907	-3.9003	2.6349	0.8462
H	5.3372	-1.5556	-1.3926	5.6438	1.4847	-2.2063	5.7136	1.9356	-1.9725
H	5.3158	-0.3849	-2.7535	5.8824	2.1350	-0.5483	5.9938	2.4129	-0.2641
H	6.5658	-0.2649	-1.4852	7.0655	0.9952	-1.2455	7.1773	1.3834	-1.1147
H	3.2197	-1.3976	-2.3082	3.6573	2.1981	-1.1565	3.7671	2.4708	-0.8423
H	-1.1138	1.5842	1.3941	-0.0433	-1.5218	1.8034	2.5936	-2.1903	2.3010
H	-0.5687	3.2672	1.6525	0.5352	-3.1757	2.1249	1.3334	-3.4102	1.9773
H	-1.0486	2.7448	0.0222	1.5432	-1.7632	2.6018	2.9388	-3.5611	1.2005
H	3.0608	3.8205	-0.0517	4.4596	-1.6294	2.3352	4.9059	-1.7648	2.1014
H	4.3594	3.6700	1.1758	5.5074	-2.8969	1.6103	5.7928	-2.9584	1.1097
H	4.6088	3.0036	-0.4608	5.7723	-1.1812	1.1911	6.1359	-1.2148	0.9189
H	-1.3831	1.3880	-1.9904	0.0237	-1.6620	-1.6637	0.2600	-1.7047	-1.4087
H	-3.6840	1.3651	-1.9014	-2.0637	-2.5630	-1.9693	-1.6633	-2.8023	-1.5775
H	-5.7797	0.7028	-2.4883	-3.5435	-4.2166	-2.1420	-2.8425	-4.6628	-1.1056
H	-7.1913	1.4060	-1.6716	-5.3003	-3.9971	-2.2473	-4.5174	-4.8465	-1.6624
H	-5.6662	2.3105	-1.6884	-4.2305	-2.6879	-2.7938	-3.4335	-3.7501	-2.5404
H	-2.4075	-2.2050	1.6238	-2.9441	0.9371	1.6503	-2.7211	0.9456	1.7556
H	-0.6964	-3.1189	0.9184	-1.3044	2.4346	1.7327	-1.3086	2.3936	1.8908
H	0.8210	-3.7103	-0.9433	0.2049	3.8977	0.5748	0.2831	3.9634	0.9300
H	-0.8411	-3.3850	-1.3811	1.0318	2.5369	1.3081	1.0882	2.5405	1.5734
H	1.1712	-2.4511	-2.8679	0.2352	2.3417	-1.6270	0.3975	2.5496	-1.4015
H	-0.3226	-1.5462	-2.6931	1.6557	3.2846	-1.1956	1.7747	3.4806	-0.8387
H	1.7309	-2.9591	0.8409	-2.4034	2.3430	-0.9315	-1.3446	3.4266	-0.8126
H	3.7396	-0.8673	1.9494	-3.7411	4.0334	-1.6182	-2.9968	4.4169	-1.7813
H	3.0874	-1.5215	3.4625	-4.5416	4.6245	-0.1405	-4.5034	3.4841	-1.6120
H	3.4081	-2.6024	2.0887	-3.3605	5.6452	-0.9534	-4.2222	4.8793	-0.5763
Li	1.9235	1.4584	2.2540	2.8389	-3.4980	0.1182	-4.9632	1.1696	1.3312

Table S9. Atomic coordinates of optimized 2:1 stoichiometry colchicine complexes with lithium cation.

2:1 stoichiometry colchicine complexes atomic coordinates						
	Type **D**			Type **E**		
Atom	x	y	z	x	y	z
C	-5.3880	-4.7528	-2.5296	-9.7495	0.2873	0.7884
O	-4.1487	-4.0739	-2.6421	-8.6686	1.1930	0.8977
C	-4.0053	-2.8977	-1.9993	-7.4584	0.7949	0.4681
C	-4.9896	-2.2916	-1.2146	-7.1817	-0.4453	-0.1048
C	-4.7333	-1.1016	-0.5323	-5.8942	-0.7780	-0.5265
C	-3.5027	-0.4514	-0.6823	-4.8399	0.1386	-0.4162
C	-2.5018	-1.0850	-1.4234	-5.1329	1.3930	0.1795
O	-1.1914	-0.6736	-1.3061	-4.1684	2.3492	0.3322
C	-0.6361	0.0498	-2.4087	-3.4696	2.2799	1.5662
C	-2.7365	-2.3000	-2.0705	-6.4095	1.7133	0.6362
O	-1.6390	-2.9562	-2.5597	-6.6167	2.8836	1.2936
C	-1.6017	-3.2491	-3.9576	-7.0767	3.9454	0.4667
C	-3.2324	0.8047	0.0691	-3.4278	-0.1032	-0.8242
C	-3.0909	1.9470	-0.6790	-2.8309	0.9328	-1.5206
C	-2.9005	3.3019	-0.3141	-1.4892	1.2349	-1.8383
C	-2.7277	3.8746	0.9195	-0.3112	0.6798	-1.3976
S	-2.4239	5.5782	1.1820	1.2815	1.2670	-1.8517
C	-2.5968	6.2628	-0.4856	0.9168	2.8678	-2.6180
C	-2.6901	3.1780	2.2222	-0.2015	-0.4710	-0.5117
O	-2.3775	3.8268	3.2232	0.9342	-0.9150	-0.2268
C	-3.0275	1.7741	2.3767	-1.3367	-1.2294	-0.0657
C	-3.2452	0.7460	1.5046	-2.6772	-1.2412	-0.3553
C	-3.5944	-0.5945	2.1511	-3.2366	-2.6705	-0.2620
C	-5.0897	-0.9176	1.9450	-4.7193	-2.9080	-0.0347
C	-5.6593	-0.6108	0.5439	-5.6414	-2.1729	-1.0101
N	-2.7772	-1.7190	1.6729	-2.8746	-3.3265	-1.5367
C	-1.4355	-1.8027	1.5412	-1.6481	-3.7755	-1.8989
C	-0.5398	-0.8191	2.2214	-1.5404	-4.3896	-3.2648
O	-0.9505	-2.7072	0.8399	-0.6619	-3.7045	-1.1616
H	-5.6016	-5.0135	-1.4852	-9.9487	0.0292	-0.2602
H	-6.2087	-4.1488	-2.9365	-9.5591	-0.6283	1.3635
H	-5.2875	-5.6671	-3.1150	-10.6175	0.7992	1.2053
H	-5.9568	-2.7715	-1.0855	-7.9737	-1.1805	-0.2212
H	0.4456	0.0762	-2.2508	-2.8689	1.3571	1.6225
H	-0.8624	-0.4549	-3.3565	-2.8058	3.1472	1.6000
H	-1.0170	1.0782	-2.4253	-4.1669	2.3207	2.4122
H	-0.5658	-3.5091	-4.1880	-8.0440	3.6952	0.0126
H	-2.2636	-4.0853	-4.1980	-7.1955	4.8175	1.1122
H	-1.8961	-2.3652	-4.5383	-6.3418	4.1688	-0.3184
H	-3.1743	1.7956	-1.7564	-3.5277	1.6752	-1.9029

H	-2.8665	3.9708	-1.1727	-1.3971	2.0862	-2.5108
H	-3.5923	6.0769	-0.9018	0.3462	3.5093	-1.9367
H	-2.4642	7.3415	-0.3719	1.8927	3.3347	-2.7810
H	-1.8252	5.8900	-1.1678	0.4066	2.7722	-3.5814
H	-3.0566	1.5282	3.4401	-0.9818	-2.0956	0.4894
H	-3.4325	-0.5005	3.2328	-2.6805	-3.1977	0.5228
H	-5.2429	-1.9780	2.1930	-4.8755	-3.9931	-0.0968
H	-5.6587	-0.3430	2.6857	-4.9780	-2.6138	0.9905
H	-6.6443	-1.0829	0.4538	-5.2202	-2.1548	-2.0279
H	-5.8219	0.4706	0.4476	-6.5935	-2.7093	-1.0897
H	-3.2581	-2.4408	1.1437	-3.6096	-3.4075	-2.2278
H	-1.0371	-0.1743	2.9499	-2.4620	-4.3429	-3.8512
H	0.2566	-1.3849	2.7174	-0.7385	-3.8811	-3.8090
H	-0.0615	-0.1967	1.4489	-1.2425	-5.4366	-3.1522
Li	-0.2597	-2.5806	-0.9247	1.1093	-2.9463	-0.6770
C	4.3912	3.4985	-4.6644	5.2632	5.5792	-1.6080
O	3.2686	3.4471	-3.8045	4.1666	5.1393	-0.8323
C	3.3293	2.6467	-2.7170	4.1940	3.8760	-0.3537
C	4.4376	1.8714	-2.3757	5.2313	2.9705	-0.5686
C	4.4016	1.0259	-1.2697	5.1535	1.6864	-0.0315
C	3.2532	0.9548	-0.4706	4.0328	1.2714	0.6945
C	2.1422	1.7481	-0.8070	3.0081	2.2122	0.9608
O	1.0064	1.5903	-0.0702	2.0021	1.8085	1.7696
C	0.4661	2.7477	0.5507	0.6674	2.2717	1.6249
C	2.1670	2.5920	-1.9243	3.0916	3.5065	0.4359
O	1.0228	3.2424	-2.2854	2.0914	4.4135	0.6536
C	1.0798	4.6650	-2.2843	2.3732	5.3444	1.6905
C	3.2051	0.0479	0.7085	3.9034	-0.1338	1.1630
C	2.9449	0.6285	1.9276	3.6717	-0.3232	2.5097
C	2.8021	0.0927	3.2282	3.3285	-1.4638	3.2728
C	2.8567	-1.2039	3.6732	2.8566	-2.6839	2.8529
S	2.5646	-1.6895	5.3289	2.2416	-3.9477	3.9059
C	2.2127	-0.1078	6.1392	2.7976	-3.3827	5.5336
C	3.1429	-2.4041	2.8628	2.7123	-3.0276	1.4457
O	3.1385	-3.5036	3.4231	1.8464	-3.8383	1.0597
C	3.4379	-2.3452	1.4436	3.6346	-2.5044	0.4671
C	3.4599	-1.3541	0.5053	4.0410	-1.2245	0.2296
C	3.8200	-1.7422	-0.9298	4.5274	-0.9041	-1.1835
C	5.2499	-1.3337	-1.2925	5.9577	-0.3553	-1.2569
C	5.5604	0.1270	-0.9440	6.2796	0.7136	-0.2196
N	3.6545	-3.1547	-1.2274	4.4984	-2.0193	-2.1372
C	2.4529	-3.7002	-1.4860	3.4243	-2.6511	-2.6718
C	2.3993	-5.1832	-1.6825	3.7153	-3.5974	-3.8041
O	1.4312	-3.0018	-1.5450	2.2732	-2.5014	-2.2594
H	5.2749	3.8919	-4.1455	6.1954	5.5648	-1.0279
H	4.6181	2.5081	-5.0799	5.3853	4.9646	-2.5101

H	4.1205	4.1745	-5.4764	5.0370	6.6057	-1.8997
H	5.3413	1.9083	-2.9787	6.1173	3.2583	-1.1287
H	1.2663	3.3968	0.9338	0.4829	3.1557	2.2453
H	-0.1413	2.3967	1.3938	0.4301	2.5127	0.5841
H	-0.1671	3.3169	-0.1400	0.0299	1.4478	1.9649
H	1.8396	5.0369	-2.9783	3.2505	5.9551	1.4446
H	0.0954	5.0102	-2.6106	1.4976	5.9915	1.7807
H	1.2857	5.0454	-1.2730	2.5445	4.8235	2.6442
H	2.8221	1.7112	1.8949	3.7413	0.5829	3.1097
H	2.5982	0.8552	3.9774	3.3228	-1.2802	4.3456
H	1.3287	0.3824	5.7163	2.5733	-4.2019	6.2212
H	2.0047	-0.3566	7.1828	3.8769	-3.2002	5.5455
H	3.0725	0.5691	6.1101	2.2561	-2.4918	5.8664
H	3.6510	-3.3551	1.0944	3.7896	-3.2278	-0.3288
H	3.1231	-1.2147	-1.5950	3.8308	-0.1351	-1.5552
H	5.9587	-1.9903	-0.7643	6.6647	-1.1906	-1.1364
H	5.3832	-1.5035	-2.3702	6.1004	0.0513	-2.2688
H	5.7953	0.2038	0.1263	6.5023	0.2314	0.7419
H	6.4588	0.4453	-1.4847	7.1920	1.2412	-0.5212
H	4.4594	-3.7657	-1.1648	5.3914	-2.2475	-2.5548
H	1.9285	-5.6325	-0.8009	3.1286	-3.2843	-4.6732
H	1.7664	-5.4105	-2.5444	4.7696	-3.6499	-4.0890
H	3.3827	-5.6400	-1.8221	3.3699	-4.5975	-3.5249

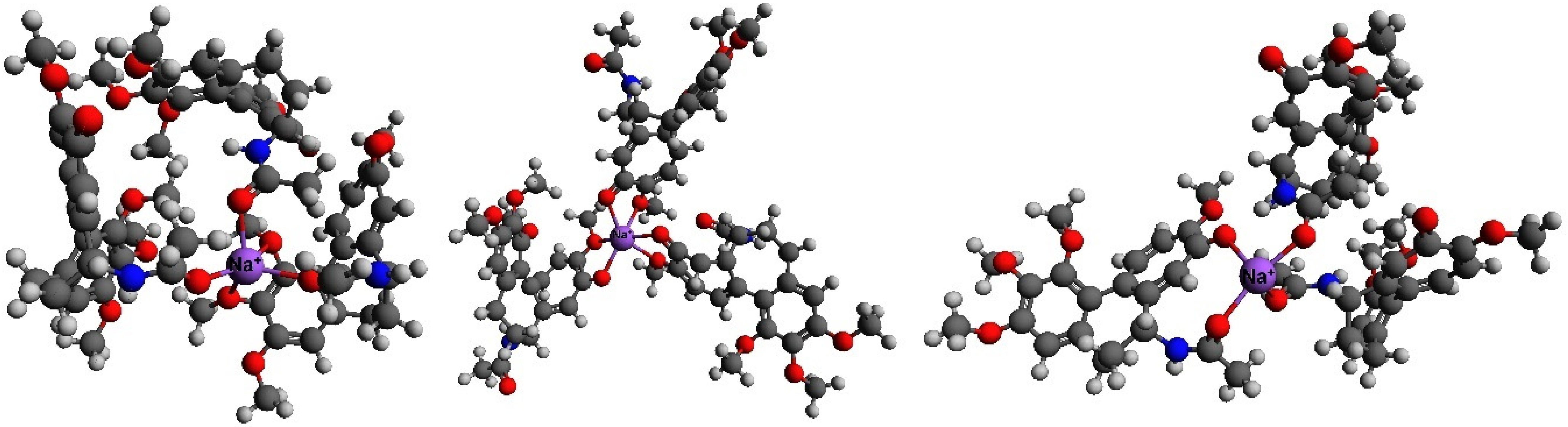

F **G** **H**

Figure S26. Optimized structures **F-H** with 3:1 stoichiometry

Figure S27. Optimized structures : **A-C** with 1:1 stoichiometry, **D** and **E** with 2:1 stoichiometry, **F-H** with 3:1 stoichiometry

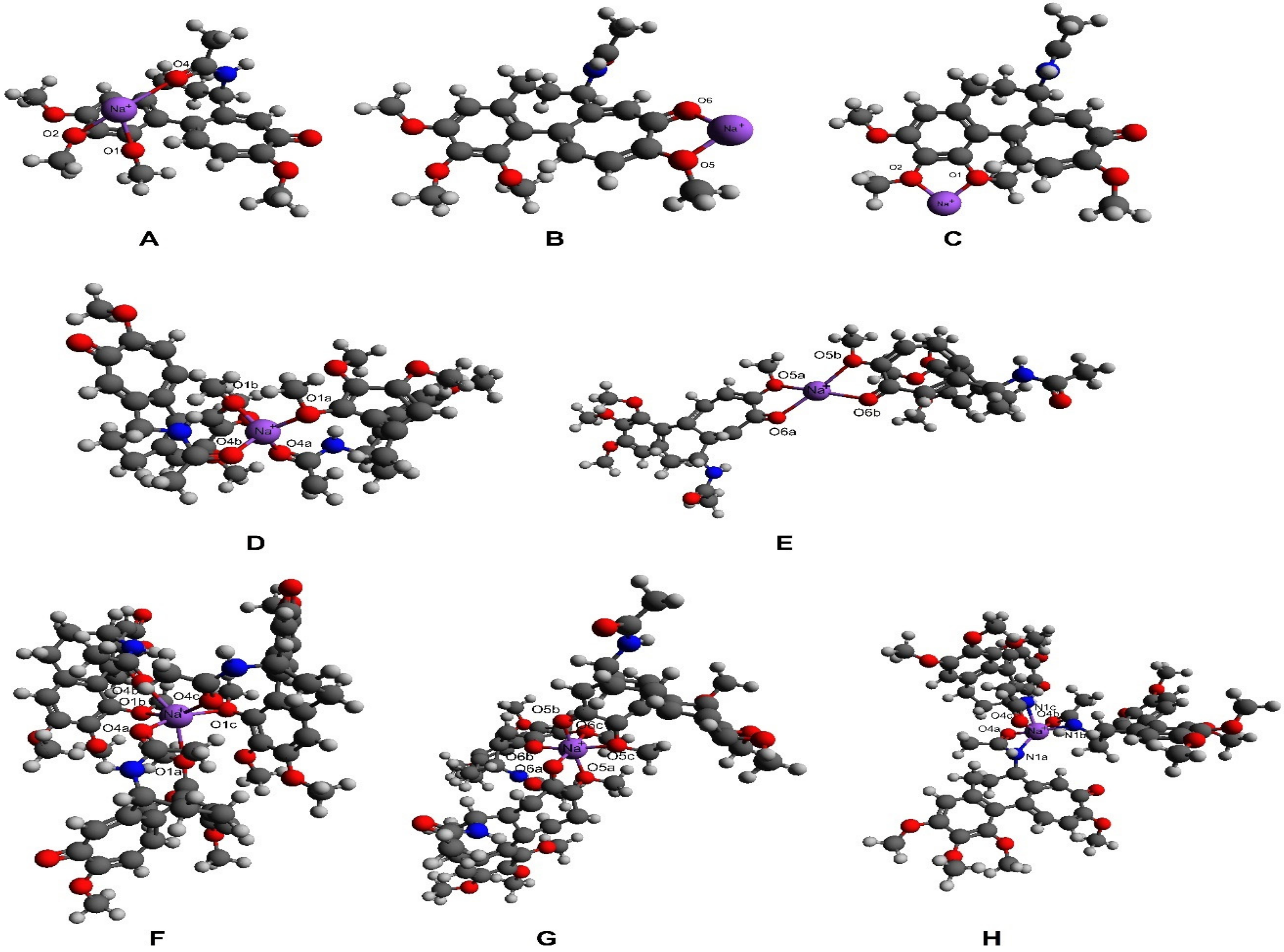

8. Impact of water solubility on the cytotoxic activity of tested compounds

Figure S28. Cytotoxic effects of tested compounds and their corresponding salts (LiCl, NaCl, KCl) on SKOV-3 cells assessed using the MTT assay. Cells were exposed to the compounds for 72 hours, after which cell viability was measured relative to the untreated control (set as 100%). Each graph shows the dose-response relationship between compound concentration (log scale) and cell viability. The names of the compounds and their respective salts are indicated on each plot. Data are presented as mean ± SD from three independent experiments.

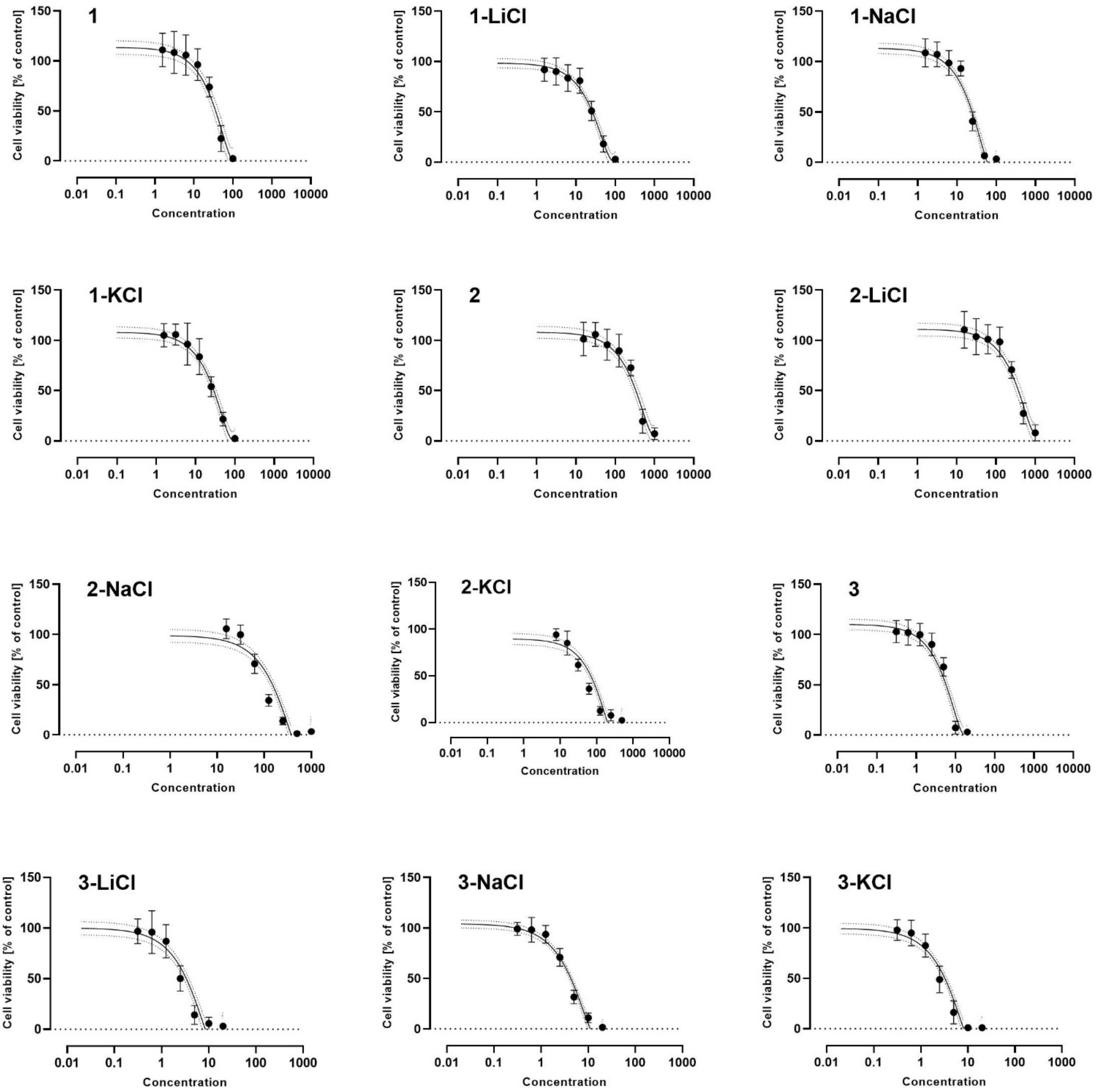

Figure S29. Impact of water solubility on the cytotoxic activity of tested compounds. r2 =0.67

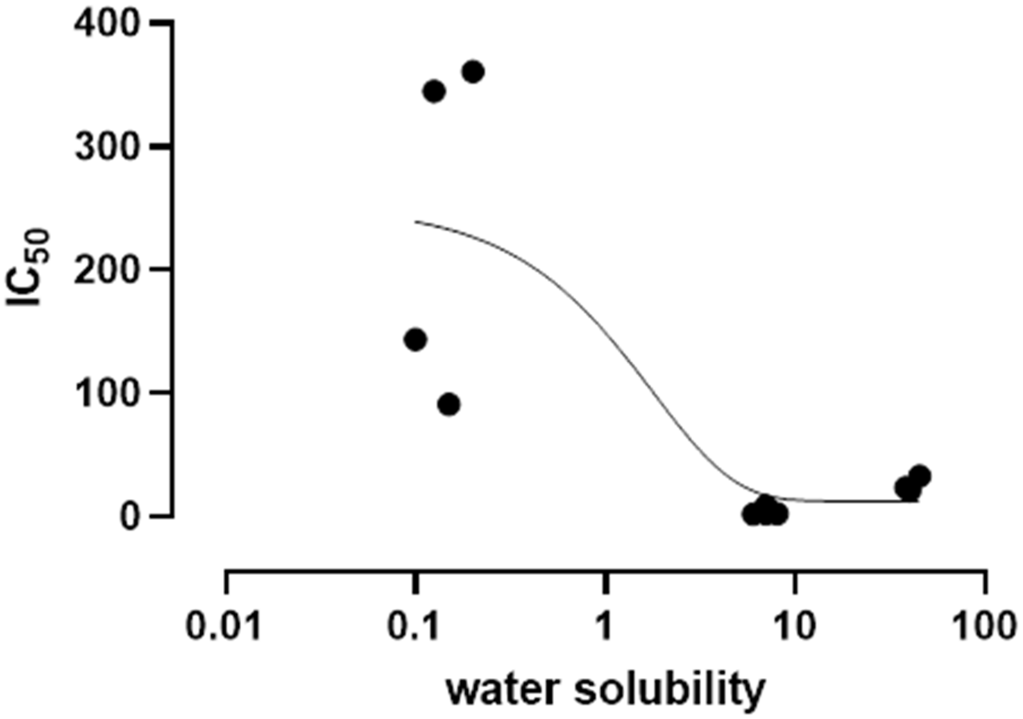

Supplement: Supplementary file 1 [file ijms-27-02985-s001.zip › ijms-4052491-supplementary.pdf]
